# Comparative Mitogenomics of Andini (Hemiptera: Cixiidae) Reveals Rapid Radiation, Clarifies Relationships, and Supports Taxonomic Revision Within the Tribe

**DOI:** 10.1002/ece3.72321

**Published:** 2025-10-20

**Authors:** Sha‐Sha Lv, Thierry Bourgoin, Xiao‐Ya Wang, Lin Yang, Yu‐Bo Zhang, Xiang‐Sheng Chen

**Affiliations:** ^1^ Guizhou Key Laboratory of Agricultural Biosecurity Guizhou University Guiyang P. R. China; ^2^ Institute of Entomology Guizhou University Guiyang P. R. China; ^3^ The Provincial Special Key Laboratory for Development and Utilization of Insect Resources of Guizhou Guizhou University Guiyang P. R. China; ^4^ Institut de Systématique, Evolution, Biodiversité (ISYEB‐UMR 7205), MMHN, CNRS, Sorbonne Université, EPHE, Université Des Antilles, Muséum National d'Histoire Naturelle Paris France; ^5^ College of Agriculture Anshun University Anshun P. R. China

**Keywords:** Andini, divergence time, mitogenome, phylogenetics, planthopper

## Abstract

Mitochondrial genomes from nine species representing three genera of the planthopper tribe Andini (Hemiptera: Cixiidae) are sequenced and analyzed, providing the first mitogenomic data for this group. The mitogenomes range from 15,415 to 15,902 bp and contain 37 typical mitochondrial genes and an A + T‐rich region, with A + T content between 73.5% and 76.0%. An inversion of *trnP* and *trnT* relative to the ancestral gene order is identified. Protein‐coding genes (PCGs) start with standard codons (ATN/GTG/TTG) and terminate with complete (TAA/TAG) or incomplete (T) stop codons. Most tRNAs display canonical cloverleaf secondary structures, except for *trnS1* in *Andes hemina*, *Parandes circinatus*, and *Parandes fuscus*, which lacks the DHU stem. Among the 13 PCGs, *cox1* shows the lowest nucleotide diversity, while *nad2* shows the highest. Ka/Ks ratios indicate purifying selection, ranging from 0.0578 (*cox1*) to 0.3080 (*nad5*). Phylogenetic analyses, based on the present sampling, support Andini and Cixiini as sister groups forming a clade sister to Pentastirini, consistent with previous studies. Within Andini, *Andixius* appears sister to a clade comprising *Andes* and *Parandes*. *Andes hemina* clusters within *Parandes*, supporting its transfer to that genus as *Parandes hemina* (Fennah 1978) **comb. nov**., based on phylogenetic and morphological evidence. Divergence‐time estimates suggest rapid diversification of Andini during the Cretaceous and Paleogene. Ancestral state reconstructions suggest that the common stalk (ScP + *R* + MP) represents a derived condition, whereas the ancestral state involves veins ScP + *R* and MP arising separately from a common point on the basal cell.

## 1 Introduction

Cixiidae Spinola, 1839, is the largest family of planthoppers (Hemiptera: Fulgoromorpha: Delphacoidea), comprising approximately 2640 described species across 256 genera, representing approximately 18.41% and 10.49% of the species and genus diversity, respectively, among all planthoppers (Bourgoin 2025). They are distributed worldwide, occurring in all zoogeographical regions, with higher species richness in the tropics (Holzinger et al. 2002; Bourgoin 2025). As obligatory phytophagous insects, cixiids maintain close associations with their host plants, which serve as sources for feeding, mating, oviposition, and communication (Claridge 1985; Wilson et al. 1994; Sforza and Bourgoin 1998; Bartlett et al. 2018). Cixiids are sap‐sucking insects whose host plants span more than 51 orders (Maixner 1994; Howard and Oropeza 1998; Sforza et al. 1998; Weber and Maixner 1998; Orenstein et al. 2003; Chen et al. 2024; Chen and Yang 2024; Bourgoin 2025; Luo et al. 2025). Several cixiids are recognized as pests of economically important crops, acting as vectors for plant pathogens such as viruses, bacterium‐like organisms, and phytoplasmas (Sforza et al. 1998; Danet et al. 2003; Jovic et al. 2007; Sémétey et al. 2007; Cui et al. 2024; Pavan et al. 2024; Pfitzer et al. 2024; Therhaag et al. 2024). They have caused significant losses to agricultural production, thereby holding considerable economic significance.

The family currently includes a single formally recognized subfamily, Cixiinae Spinola, 1839, and 19 tribes. However, ongoing studies involving molecular analyses indicate that the current classification does not accurately reflect the evolutionary history of the family, within which four major lineages are currently recognized: the borystheninian, oecleinian, pentastirinian, and cixiinian lineages (Luo et al. 2021, 2025; Bourgoin et al. 2023; Bucher et al. 2023).

**FIGURE 1:**
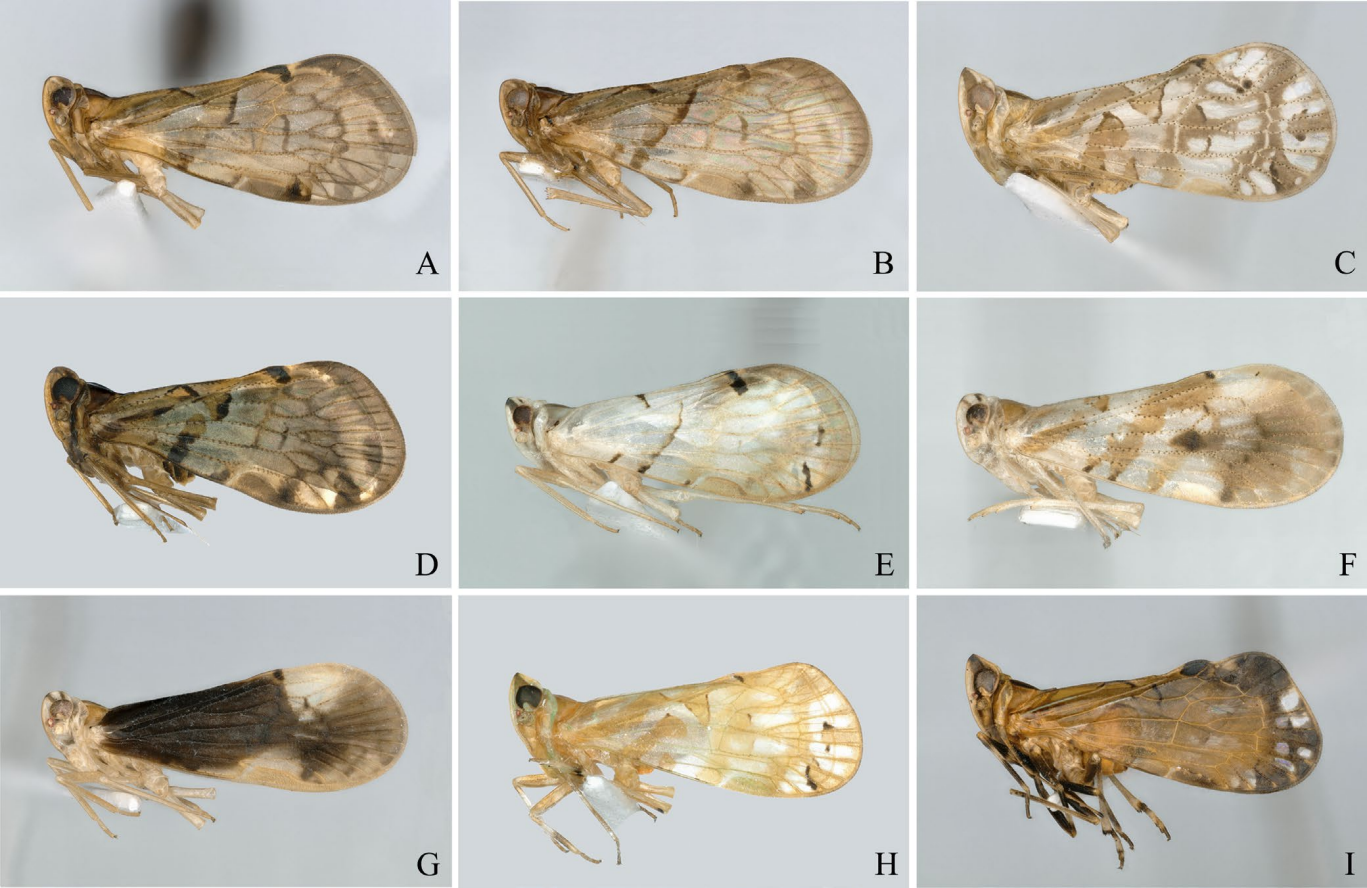
Representative species of Andini. (A) *Andes bifidus*, (B) *Andes furcutus*, (C) *Andes hemina*, (D) *Andes latanalus*, (E) *Andes pallidus*, (F) *Andixius cultratus*, (G) *Andixius truncatus*, (H) *Parandes circinatus*, (I) *Parandes fuscus*.

Within the Cixiinae, the tribe Andini—erected by Emeljanov in 2002—constitutes a small group that is neither present in the Nearctic nor in the Neotropical regions and belongs to the broader cixiinian lineage (Emeljanov 2002; Luo et al. 2025). It currently comprises 138 species across three genera (Figure 1): *Andes* Stål, 1866, *Parandes* Muir, 1925, and *Andixius* Emeljanov & Hayashi, 2007. The genus *Andes* is the most species‐rich, representing approximately 88% of the tribe's species worldwide (Wang et al. 2020; Bourgoin 2025). In China, only 29 species of Andini occur, 15 assigned to *Andes*, nine to *Andixius*, and five to *Parandes* (Fennah 1956; Zhi et al. 2018; Wang et al. 2020, 2022; Wang, Zhi, Yang, and Chen 2023; Wang, Zhi, Yang, Long, et al. 2023; Luo et al. 2022; Bourgoin 2025; Lv et al. 2025). The main distinguishing features of this tribe include: frons with median carina not bifurcated; mesonotum with three distinct longitudinal carinae; forewing strongly tectiform, membranous part of clavus protruding at the posterior part, tubercles distributed only along veins, ScP + *R* and MP forming common points or a short common stalk near basal cell; MP and CuA1 of hindwing with punctate anastomosis or V‐type (Le Cesne et al. 2022). The metatibia carries spiniform sensilla ranging from small to medium in size, and with six apical teeth, without diastema. Metatarsomere I displays eight apical teeth and is devoid of dorsal sensilla, whereas metatarsomere II bears seven or eight apical teeth and exhibits a subdorsal acutellar sensilla (Brożek et al. 2024).

The host plant relationships for Andini species have not yet been determined. However, field observations suggest ecological preferences, with *Andes* and *Andixius* being mostly found in dark and humid environments rich in mosses and ferns, whereas *Parandes* was collected from arboreal habitats.

Currently, there are very few reports on the mitochondrial genomes of the entire Cixiidae family. Only a few valid datasets can be retrieved from the NCBI database, and some entries are less than 10 kb and considered invalid. No mitochondrial genetic data are available for the tribe Andini. Investigations of the Andini have predominantly revolved around new species discovery and formal description, with relatively few studies on DNA barcoding. To date, no comprehensive study has been conducted specifically on the phylogenetic relationships within the Andini tribe. The tribe has only been briefly mentioned in broader phylogenetic analyses of the Cixiidae family (Emeljanov 2002; Ceotto and Bourgoin 2008; Ceotto et al. 2008; Bucher et al. 2023; Deng et al. 2025; Luo et al. 2025).

The insect mitochondrial genome is a compact, circular, double‐stranded DNA molecule that houses 37 genes: 13 protein‐coding genes (PCGs), 22 transfer RNA genes (tRNAs), the small and large ribosomal RNAs (rRNAs: *rrnS* and *rrnL*), and a noncoding AT‐rich control region (Boore 1999; Cameron 2014). Its compactness, maternal inheritance, conserved gene repertoire, accelerated evolutionary rate, and rarity of recombination have made the mitogenome a favored tool for exploring insect phylogenetic relationships, conservation genetics, molecular evolution, phylogeography, and population genetics (Ballard and Whitlock 2004; Bjork et al. 2011; Foote et al. 2011; Crampton‐Platt et al. 2015; Timmermans et al. 2016).

Here we present the first mitochondrial assemblies for nine Andini representatives. We analyzed genome organization and composition, PCGs and codon usage, ribosomal and transfer RNA genes, overlapping and intergenic spacer regions, nucleotide diversity, and evolutionary rates. We also built a phylogenetic tree incorporating seven additional Cixiidae species from public databases, and estimated divergence times and ancestral character states. With this work, we provide new insights into the mitochondrial genome structure and intergeneric phylogenetic relationships within Andini, and we want to contribute to broader systematic and evolutionary studies of Cixiidae and planthoppers in general.

## 2 Materials and Methods

### 2.1 Morphological Treatment

Specimens were collected using sweeping methods. No specific permits were required for the collection of these Andini species, as they are not endangered or protected in China. Terminology of external morphology follows Bourgoin (1987) for male genitalia and Bourgoin et al. (2015) for wing venation. Dry specimens were used for the descriptions and illustrations. Body measurements are from the apex of the vertex to the tip of the forewing; vertex length (median length of the vertex) was measured from the apical transverse carina to the tip of the basal emargination. Photographs for adult habitus were obtained by the KEYENCE VHX‐6000 system, and multiple layers were stacked using Helicon Focus 6. External morphology and drawings were done under the Leica MZ 12.5 stereomicroscope. The photographs and illustrations were scanned with a CanoScan LiDE 200 and imported into Adobe Photoshop 6.0 for labeling and plate composition.

The specimens examined are deposited in the Institute of Entomology, Guizhou University, Guiyang, Guizhou Province, China (IEGU).

### 2.2 Sample Preparation and DNA Extraction

Nine Andini species belonging to three genera (Figure 1) were gathered from diverse localities across China (Table S1). All specimens were fixed in absolute ethanol upon collection and subsequently stored at −40°C in a laboratory refrigerator of the Institute of Entomology, Guizhou University (IEGU). After morphological identification, thoracic and leg muscle tissue was dissected from each specimen. Total genomic DNA was extracted using the DNeasy DNA Extraction kit (Qiagen, Hilden, Germany), and its integrity and purity were verified by 1% agarose electrophoresis. DNA concentration and purity were determined with a NanoDrop spectrophotometer (Thermo Fisher Scientific, Waltham, MA).

### 2.3 Mitogenome Sequencing and Assembly

The sequences of the mitochondrial genome were generated via next‐generation sequencing technologies. For every specimen, paired‐end libraries with 350‐bp inserts were constructed following Illumina's standard protocol. Library quality was assessed by qPCR and the Agilent 2100 Bioanalyzer (Agilent Technologies, USA). Libraries meeting quality thresholds were sequenced on an Illumina HiSeq 4000 system (Illumina, San Diego, CA) using the PE150 strategy, yielding ≥ 2 Gb of raw data per sample. Sequencing datasets were assembled with MitoZ v2.4‐alpha (Meng et al. 2019) under default settings, using the published mitogenome of *Oecleopsis sinicus* (Fulgoroidea: Cixiidae; NC_066441) as the reference.

### 2.4 Mitogenome Annotation and Analysis

Assembled mitogenomes were loaded into Geneious Prime 2021.1.1 (Kearse et al. 2012) for gene annotation. tRNA genes and their secondary structures were predicted by the MITOS webserver (Bernt et al. 2013) and ARWEN v1.2 (Laslett and Canbäck 2007), both employing the invertebrate codon predictors. Open‐reading‐frame scanning under the same code pinpointed the 13 PCGs, whereas rRNAs and the AT‐rich region were delimited through homology searches against related cixiid mitogenomes.

Circular genome maps were generated with OrganellarGenomeDRAW (OGDRAW) v1.3.1 (Greiner et al. 2019). Nucleotide composition and relative synonymous codon usage (RSCU) were computed with PhyloSuite v1.2.2 (Zhang et al. 2020), and the resulting RSCU heatmaps were produced via the Genepioneer Cloud Platform (http://cloud.genepioneer.com). AT and GC skew values were computed as (A − T)/(A + T) and (G − C)/(G + C), respectively (Perna and Kocher 1995). Overlapping regions and intergenic spacers were manually curated. Concatenated alignments of the 13 PCGs were imported into DnaSP v6.12.03 (Librado and Rozas 2009) to quantify nucleotide diversity as well as nonsynonymous substitutions (Ka) and synonymous substitutions (Ks) values. The nine newly sequenced mitogenomes were deposited in GenBank under the accession numbers listed in Table 1.

**TABLE 1:** Mitochondrial genomes used in this study.

Family	Subfamily	Tribe	Organism	Length	AT%	ID	References
Cixiidae	Cixiinae	Andini	*Andes bifidus*	15,783	74.8	PV019484	This study
			*Andes furcutus*	15,677	73.5	PV019485	This study
			*Andes hemina*	15,834	74.8	PV019486	This study
			*Andes latanalus*	15,717	74.8	PV019487	This study
			*Andes pallidus*	15,578	76.0	PV019489	This study
			*Andixius cultratus*	15,415	73.7	PV019490	This study
			*Andixius truncatus*	15,902	74.2	PV019492	This study
			*Parandes circinatus*	15,445	73.7	PV019493	This study
			*Parandes fuscus*	15,757	75.8	PV019494	This study
		Cixiini	Cixiidae sp.	16,344	77.5	OR062441	Wang, Jiang, et al. (2024) and Wang, Meng, et al. (2024)
			Cixiini sp.	15,988	77.2	OR062442	Wang, Jiang, et al. (2024) and Wang, Meng, et al. (2024)
			*Iolania perkinsi*	14,949	78.7	MZ748292	Chong et al. (2022)
		Pentastirini	*Oecleopsis sinicus*	15,196	78.3	NC066441	Unpublished
			*Oecleopsis* sp.	15,382	77.1	OR062443	Wang, Jiang, et al. (2024) and Wang, Meng, et al. (2024)
			*Oliarus filicicola*	15,568	78.8	MZ748293	Chong et al. (2022)
		Borysthenini	*Borysthenes* sp.	15,297	73.3	OR062440	Wang, Jiang, et al. (2024) and Wang, Meng, et al. (2024)

### 2.5 Phylogenetic Analysis

Nine Andini mitogenomes generated in this study were combined with seven outgroup sequences downloaded from GenBank (Table 1) for phylogenetic analyses. Nucleotide sequences of the 13 PCGs were aligned independently using the MAFFT plug‐in in PhyloSuite v1.2.2 (Zhang et al. 2020), and the resulting alignments were merged into a single supermatrix using SequenceMatrix v1.7 (Vaidya et al. 2011). Optimal partitioning strategies and nucleotide‐substitution models for both maximum likelihood (ML) and Bayesian inference (BI) were identified with PartitionFinder v2.1.1 (Lanfear et al. 2017) under the greedy search algorithm, and the selected schemes are listed in Table S2.

ML analysis was carried out in IQ‐TREE v1.6.3 (Nguyen et al. 2015), applying 10,000 ultrafast bootstrap replicates. BI analysis was executed in MrBayes v3.2 (Ronquist et al. 2012) using four simultaneous Markov chains run for two million generations, sampling every 1000 generations and discarding the first 25% as burn‐in. Convergence was confirmed by effective sample sizes (ESS) > 200 in Tracer v1.7 (Rambaut et al. 2018). The consensus phylogenies were imported into FigTree v1.4.4 for visualized and edited.

### 2.6 Divergence‐Time Estimates

Divergence‐time estimation was inferred in MCMCTree based on the BI tree (PAML v4.9j; Yang 2007) using the nucleotide sequences of the 13 PCGs. Settings were clock = 1, model = 4, burn‐in = 125,000, sampfreq = 2, and nsample = 500,000. Minimum‐age calibrations were based on two fossil taxa: *Barremixius petrinus* (Fennah, 1961) at 121.4–125.77 Ma and *Acrotiara multigranulata* Luo & Bourgoin, 2021 at 98.8 ± 0.6 Ma (Luo et al. 2021, 2025). Two independent runs with different random seeds were compared for consistency, and acceptance rates were maintained around 0.3 as recommended in the manual. The final chronogram, including 95% highest posterior density (HPD) intervals, was visualized in FigTree v1.4.4 and refined in Adobe Illustrator CC 2019.

### 2.7 Ancestral State Reconstructions

**FIGURE 2:**
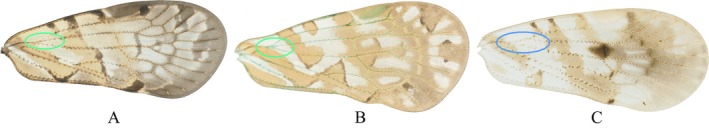
Forewings with ScP + *R* and M veins of three genera in Andini. (A) *Andes*, (B) *Parandes*, (C) *Andixius*. Green circle (common points) and blue circle (common stalk).

Using the time‐calibrated phylogenetic tree produced in the previous step, ancestral states were inferred under likelihood frameworks implemented in Mesquite v3.51 (Maddison and Maddison 2018). In Andini, a key distinguishing feature is that the ScP + *R* and MP veins of the forewing form either a common point or a short common stalk near the basal cell. This trait was encoded as a binary character, with the states defined as follows: green circle (common points) and blue circle (common stalk) (Figure 2). Proportional likelihoods were reported for ancestral node reconstructions.

## 3 Results

### 3.1 Genome Organization and Composition

**FIGURE 3:**
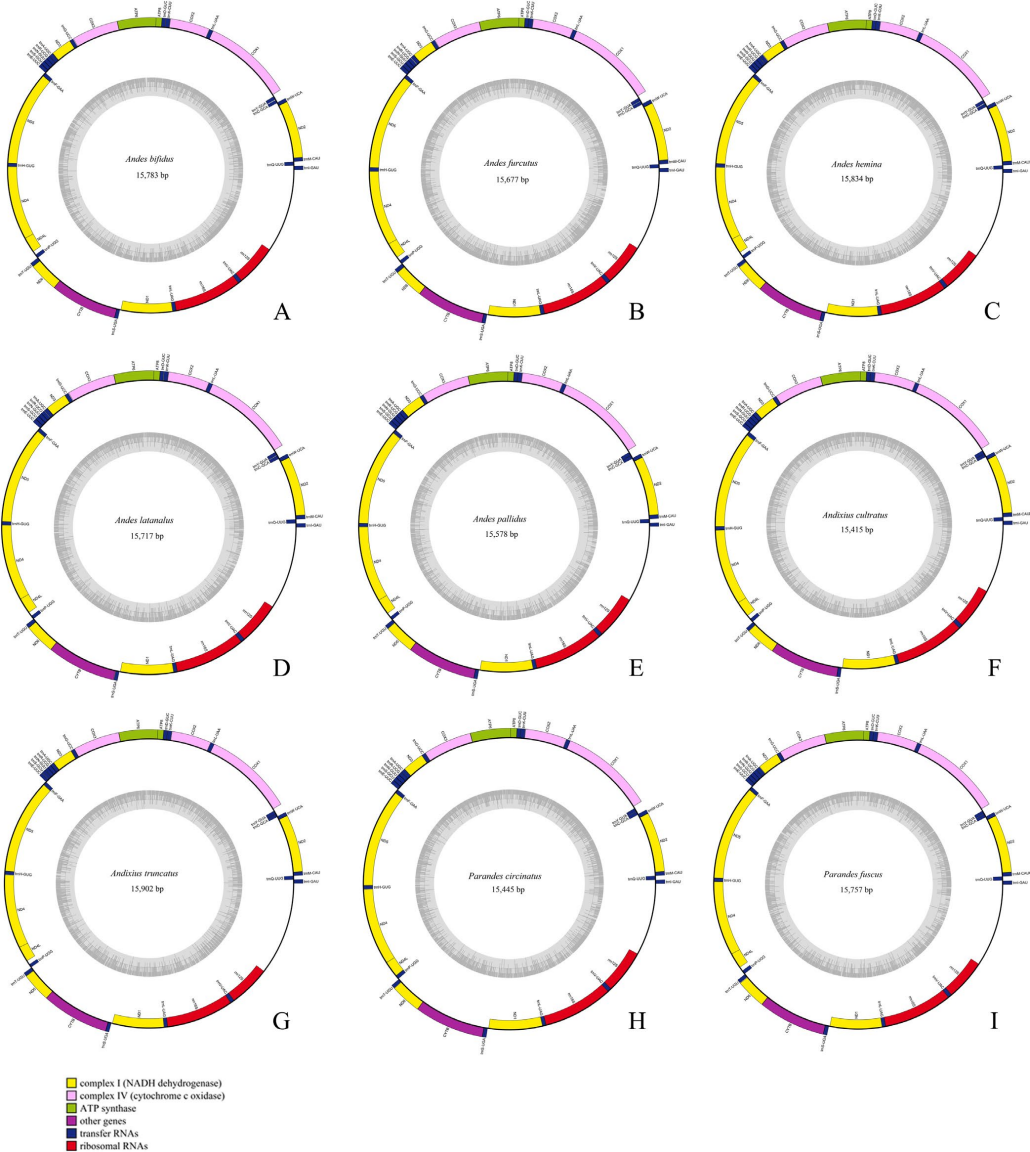
Circular maps of the mitogenomes of Andini. (A) *Andes bifidus*, (B) *Andes furcutus*, (C) *Andes hemina*, (D) *Andes latanalus*, (E) *Andes pallidus*, (F) *Andixius cultratus*, (G) *Andixius truncatus*, (H) *Parandes circinatus*, (I) *Parandes fuscus*. Color blocks outside the circle indicate that the genes are on the heavy strand (H‐strand); those within the circle indicate the genes are located on the light strand (L‐strand).

New mitochondrial genomes were obtained for nine Andini species, with assembled lengths spanning 15,415 bp in *Andixius cultratus* to 15,902 bp in *Andixius truncatus* (Figure 3 and Table 1). Compared with the putative ancestral gene order, the positions of *trnP* and *trnT* appear to be inverted. Each newly sequenced mitogenome harbors the canonical set of 37 mitochondrial genes (Figure 3 and Table S3). Across the nine mitogenomes, base composition is strongly AT‐biased, ranging from 73.5% (*Andes furcutus*) to 76.0% (*Andes pallidus*) (Table S4), and all mitogenomes also exhibit a pronounced positive AT skew (0.1590 to 0.2266) alongside a negative GC skew (−0.3387 to −0.2778).

### 3.2 PCGs and Codon Usage

Across the nine Andini mitogenomes, the concatenated 13 PCGs span 10,827–10,833 bp, and their average AT content varies from 72.0% to 74.5%. The AT skews ranging from −0.1761 to −0.1556 and GC skews from −0.0929 to −0.0593 of the PCGs were nearly identical among the nine species (Table S4). Among these PCGs, *nad5* (1690 bp) was the longest, while *atp8* (102 bp) was the shortest (Table S3). The start codons of 13 PCGs were predominantly ATN in all nine mitogenomes, with the following exceptions: *nad5* used GTG in the genus *Andes* (except for *Andes hemina*, which used TTG); *Andixius cultratus* and *Andixius truncatus* used ATC and ATT, respectively; and the two species of *Parandes* used TTG (Table S5). The stop codons of the PCGs exhibited the following patterns: the genes of *nad2*, *cox1*, *atp8*, *cox3*, and *nad4l* all used TAA; *cox2*, *atp6*, *nad5*, *nad6*, and *nad1* genes all used incomplete T as stop codons; in *nad3*, six species used TAG as the stop codon, while the remaining species (*A. hemina*, *P. circinatus*, and *P. fuscus* ) used TAA; in *nad4*, all species except those in the genus *Andixius* used an incomplete T as the stop codon; in *cytb*, all species except *Andes latanalus* (which used TAA) used TAG as the stop codon (Table S5).

**FIGURE 4:**
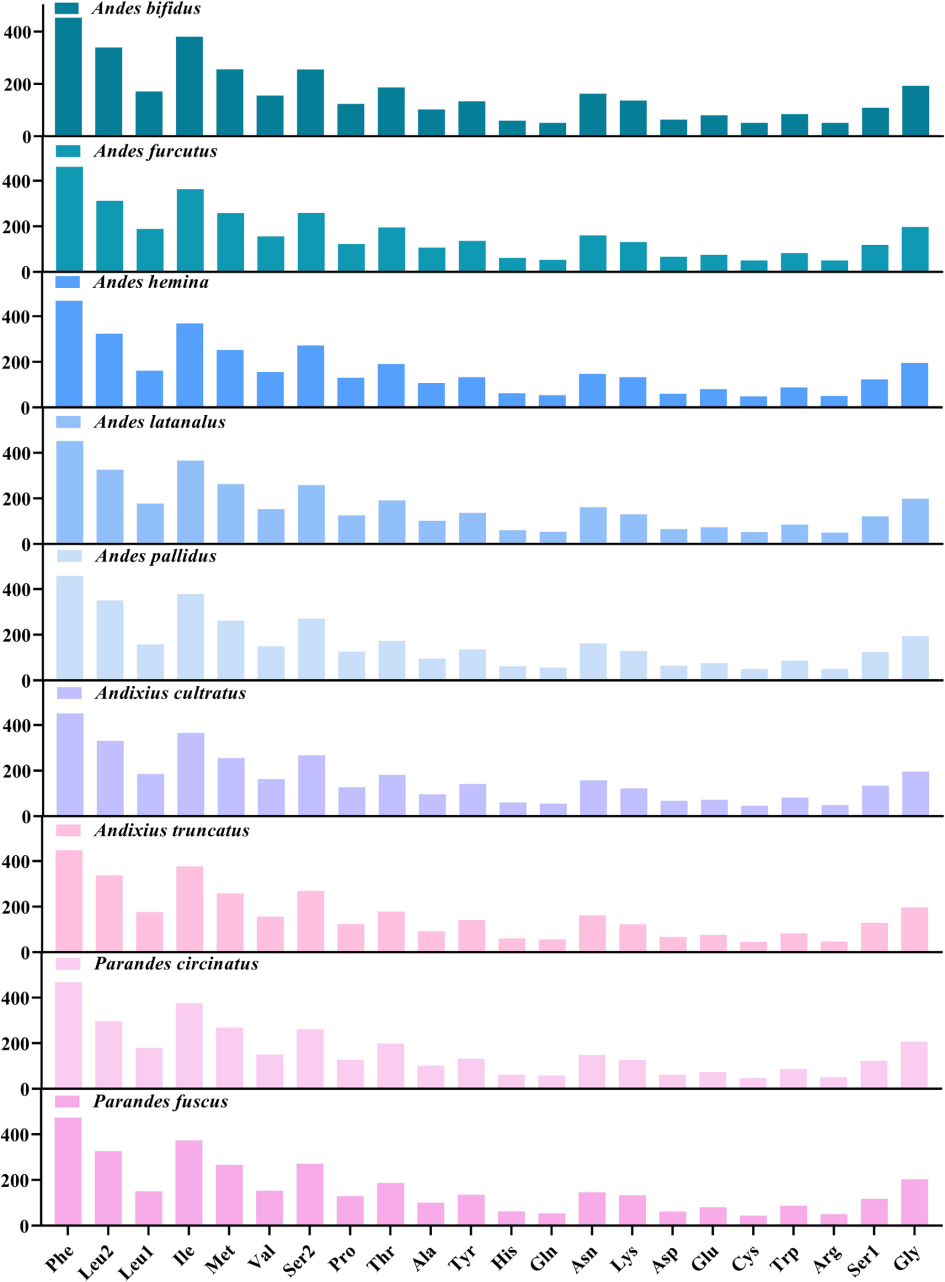
Number of codon usages in the protein‐coding genes of the nine newly sequenced mitogenomes of Andini.

**FIGURE 5:**
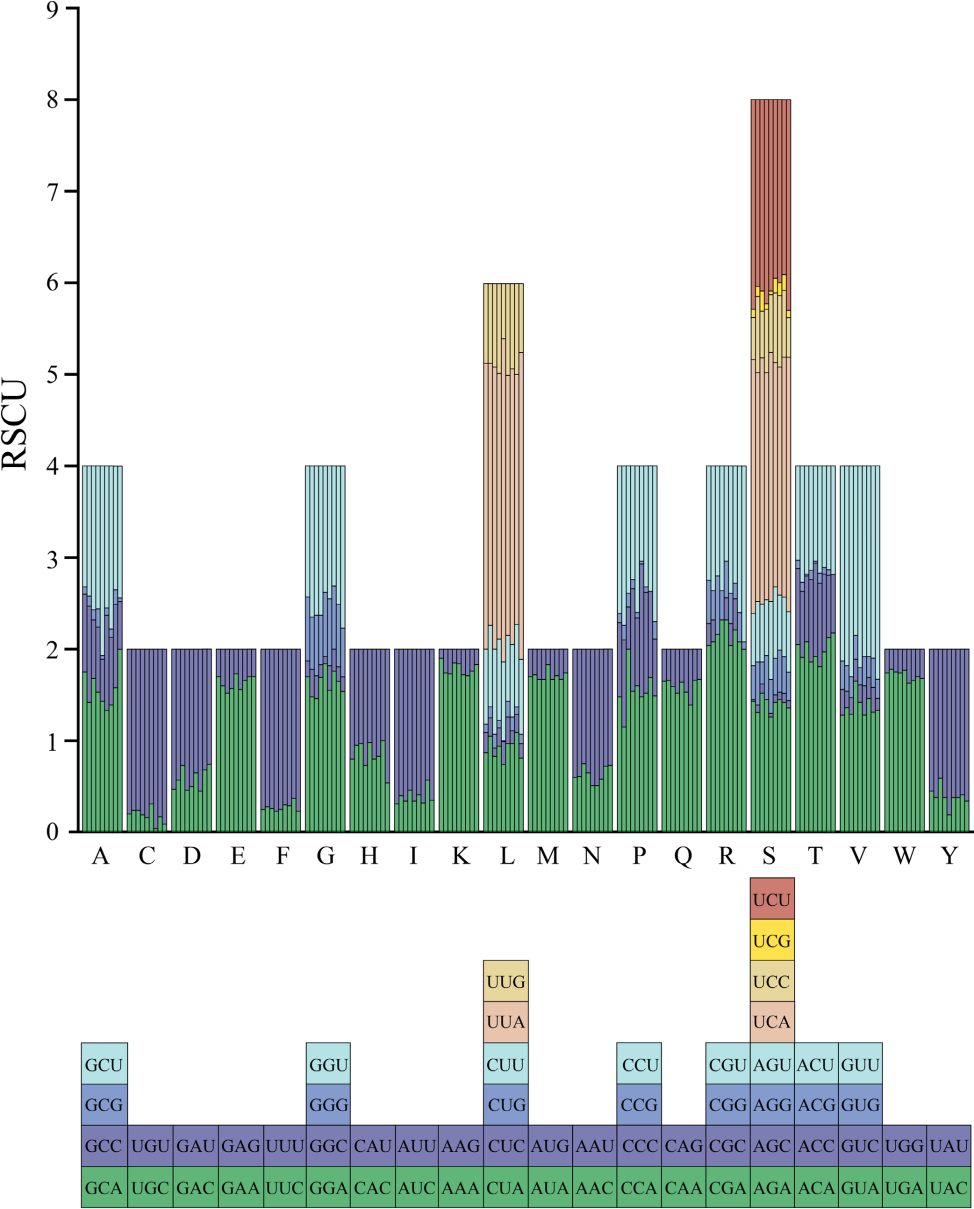
RSCU results of nine Andini. The chart from left to right was derived from nine species: *Andes bifidus*, *Andes furcutus*, *Andes hemina*, *Andes latanalus*, *Andes pallidus, Andixius cultratus*, *Andixius truncatus*, *Parandes circinatus*, and *Parandes fuscus*. The color of the columns was corresponded to the codon labeled below the abbreviated amino acids.

The codon usage patterns, RSCU values, and codon frequencies in the PCGs of the nine mitogenomes were determined (Figures 4 and 5 and Table S6). The analysis of PCGs codon usage revealed similar trends across nine species, with the codons Phe‐UUU, Ile‐AUU, and Leu2‐UUA dominating the amino acid landscape, jointly exceeding 50% of codon assignments. A pronounced A/T preference was evident in every RSCU distribution, and the overwhelming A + T load within the coding regions underpins the overall A + T richness of the entire mitogenome.

**FIGURE 6:**
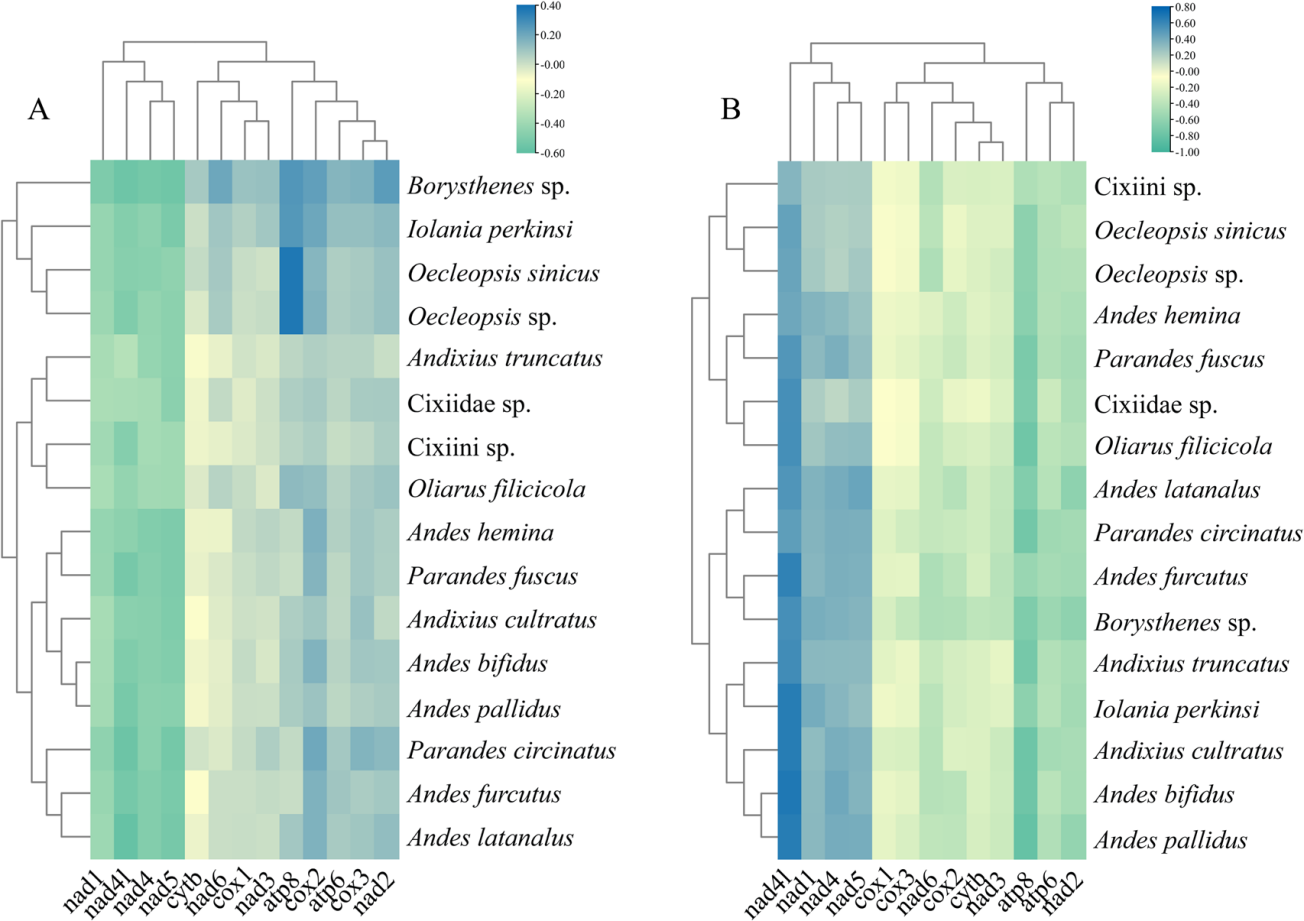
AT (A) and GC (B) skews of 13 PCGs from cixiid mitogenomes.

Across the 16 cixiid mitogenomes, we examined strand‐specific compositional bias for the 13 PCGs (Figure 6 and Table S7). On the L‐strand, AT skews were strongly negative, whereas GC skews were positive—only *nad4l* showed an exceptionally high GC skew. In contrast, H‐strand AT skews oscillated but leaned toward positive values, while GC skews remained consistently negative. Overall, A and C bases outnumbered T and G bases.

### 3.3 Ribosomal RNA and Transfer RNA Genes

The *rrnS* and *rrnL* genes were identified in all nine newly sequenced mitogenomes (Figure 3 and Table S3). Across these mitogenomes, *rrnL* length varied from 1193 bp (*Andixius truncatus*) to 1211 bp (*Parandes fuscus*), whereas *rrnS* ranged from 719 bp (*Andixius cultratus*) to 750 bp (*Andes pallidus*). The total length of rRNAs across the nine sequenced mitogenomes spanned 1918–1953 bp (Table S4). AT content of these rRNA genes varied from 72.8% to 76.8%, with AT skew values ranging from −0.2691 to −0.1209 and GC skew values ranging from 0.3162 to 0.4330.

All 22 tRNAs were recovered from the nine mitogenomes (Tables S3 and S4), spanning 1396–1421 bp in aggregate. The length of each individual tRNA gene varied between 59 and 71 bp. These tRNA genes exhibited high AT content (75.2%–77.3%) and displayed positive skews for both AT and GC. All tRNAs except *trnS1* in *Andes hemina*, *Parandes circinatus*, and *Parandes fuscus*—whose DHU stem is unstable—adopted the standard cloverleaf fold in the nine mitogenomes (Figures S1–S9). The anticodons match those universally reported for Hemiptera. Mismatches were observed in several tRNA stems and loops; six types were detected—G‐U, U–U, A‐G, A‐C, A‐A, U‐C, plus an extra adenine—with G‐U pairings being the most abundant.

### 3.4 Overlapping and Intergenic Spacer

Across the nine mitogenomes were detected, 8–13 intergenic spacers were detected (1–75 bp; Table S3); the longest (75 bp) lies between *nad4l* and *trnP* in *Andes pallidus*. Additionally, the nine species exhibited 9–14 overlapping genes, with overlap lengths varying from 1 to 11 bp. Among the nine newly sequenced mitogenomes, four gene overlaps were conserved: *trnW*–*trnC* (8 bp: AAGCCTTA), *cox1*–*trnL2* (5 bp: TCTAA), *atp8*–*atp6* (11 bp: ATA(T)C(T)TAACTAA), *nad4*–*nad4l* (7 bp: TTAACAT).

### 3.5 Nucleotide Diversity and Rate Assessment

**FIGURE 7:**
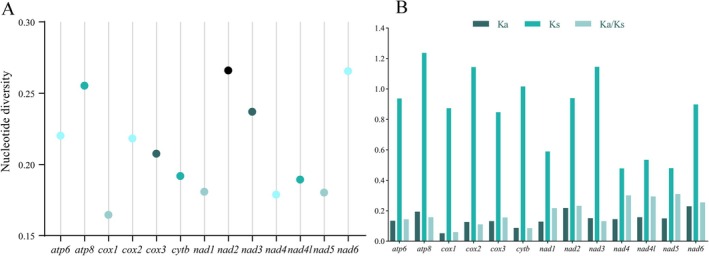
Nucleotide diversity (A) and the ratio of Ka/Ks (B) of protein‐coding genes from nine newly sequenced Andini mitogenomes.

To quantify the evolutionary rate of mitochondrial 13 PCGs across the nine Andini mitogenomes, we estimated nucleotide diversity, Ka, Ks, and their ratio (Ka/Ks) for every aligned PCG. The nucleotide diversity values for individual genes spanned 0.1646 (*cox1*) to 0.2659 (*nad2*) (Figure 7A and Table S8). *nad2*, *nad6*, and *atp8* exhibited high levels of nucleotide diversity, indicating significant variability. In contrast, *cox1* and *nad4* displayed relatively conserved levels of nucleotide diversity.

To assess the selection pressure acting on the mitochondrial PCGs, the ratio of Ka/Ks was calculated for the 13 PCGs in the mitogenomes of the nine species. All 13 PCGs exhibited Ka/Ks ratios well below 1, spanning 0.0578 (*cox1*) to 0.3080 (*nad5*) (Figure 7B and Table S9). These results indicate that all 13 PCGs were under purifying selection. The relative evolutionary rates of the 13 PCGs were ranked as follows: *nad5* > *nad4* > *nad4l* > *nad6* > *nad2* > *nad1* > *atp8* > *cox3* > *atp6* > *nad3* > *cox2* > *cytb* > *cox1*. Within this spectrum, *cox1* is subject to the strongest purifying selection, whereas *nad5* experiences the weakest selective pressure and evolves most rapidly among the Andini PCGs.

### 3.6 Phylogenetic Relationships

**FIGURE 8:**
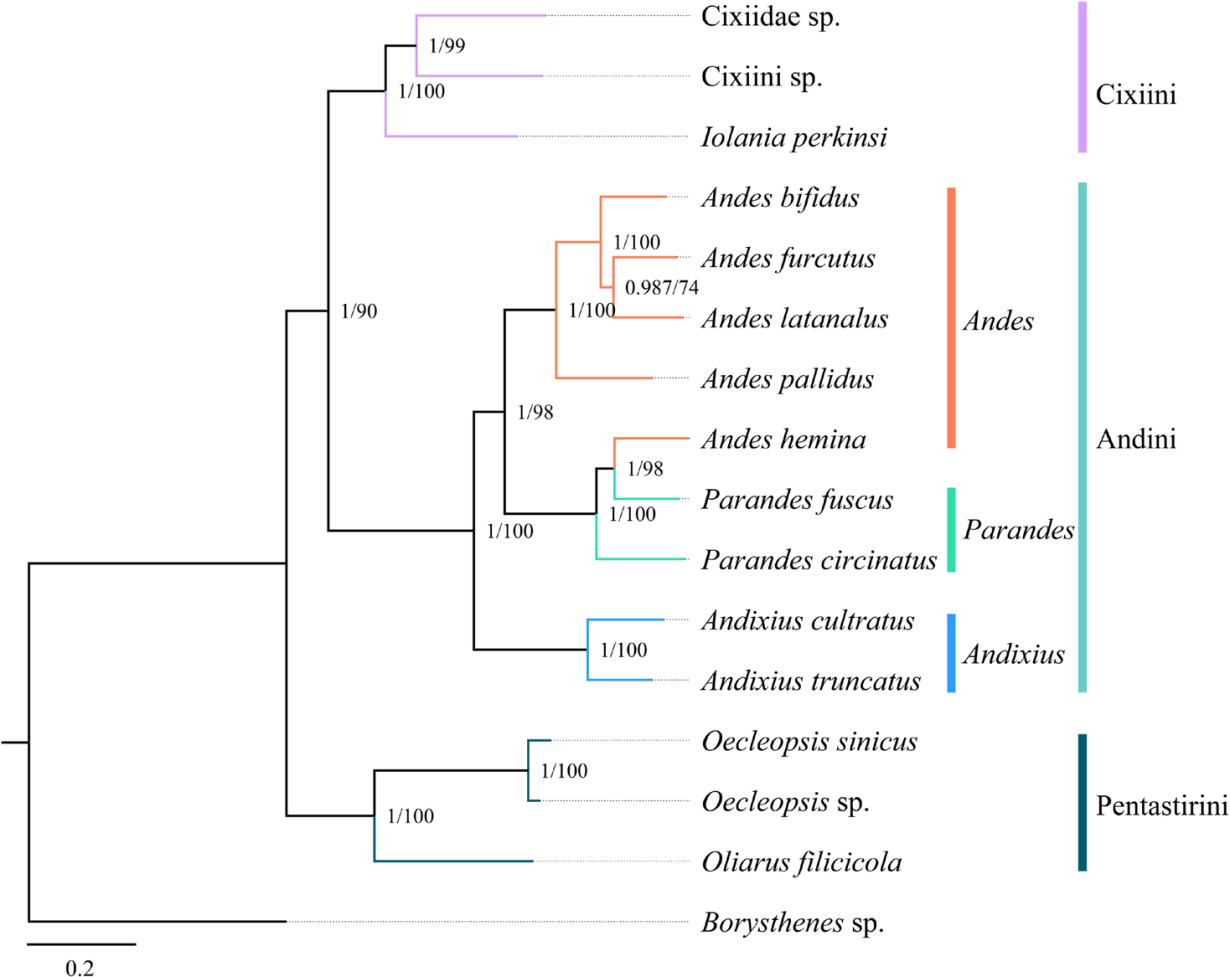
Phylogenetic trees of Cixiidae were inferred using MrBayes (Bayesian inference) and maximum likelihood (ML) analysis based on the nucleotide sequences of the 13 protein‐coding genes. Bayesian posterior probabilities (BPP) and bootstrap percentages (BP) are indicated on branches.

Phylogenetic analyses were conducted on 16 species from the family Cixiidae, including nine species of Andini and seven other cixiids, using concatenated nucleotide sequences of 13 PCGs by applying both BI and ML approaches. The resulting phylogenetic trees (Figure 8) exhibited largely consistent topologies with high node support values. In these phylogenetic trees, all cixiid species were well clustered and divided into three main clades representing the usual tribes Pentastirini, Andini, and Cixiini, with Pentastirini robustly supported (BPP = 1; BP = 100), sister to a clade (Andini + Cixiini) (BPP = 1; BP = 90).

In clade of Andini, *Andes hemina* (formerly assigned to *Andes*) clustered with *Parandes*, and three main clades are observed. *Andixius* genus and its two species is positioned at the base, sister to (*Andes* + *Parandes*), formed a strongly supported monophyletic group (BPP = 1; BP = 100). In *Parandes*, *P. circinatus* finds a sister position to ( *P. fuscus* + *A. hemina*) while in *Andes*, *A. pallidus* is sister to ( *A. bifidus* + (*A. latanalus* + *A. furcutus*)).

### 3.7 Divergence‐Time Estimates

**FIGURE 9:**
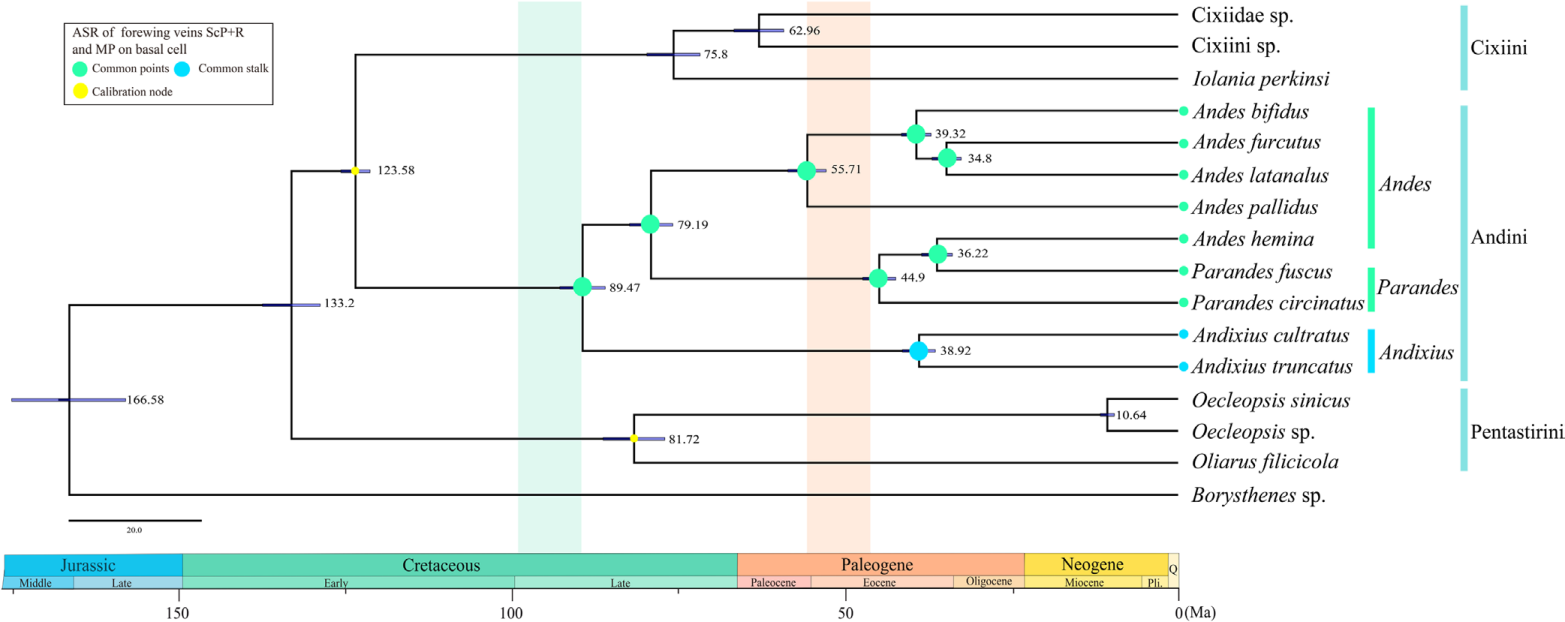
Chronogram showing the temporal divergences and ancestral state reconstructions of Cixiidae. Blue bars indicate time intervals for 95% probability of actual age (Table 2). Pie charts on nodes indicate most likely states only. The color of nodes at end of the tree correspond to states of forewing veins ScP + *R* and M of Andini on the left box of the figure. Calibration nodes by the yellow dot.

The results of divergence‐time analysis are illustrated in Figure 9 and Table 2 and place the onset of Pentastirini radiation in the Late Cretaceous (~81.72 Ma; 95% HPD: 77.12–86.39 Ma). The Andini‐Cixiini split is dated to the Early Cretaceous (~123.58 Ma; 95% HPD: 121.4–125.77 Ma), whereas diversification within Andini commenced in the Late Cretaceous (~89.47 Ma; 95% HPD: 86.07–92.89 Ma).

**TABLE 2:** Estimation of diversification times (Ma) for major groups in this study.

Clade	Mean	Period	95% HPD
Pentastirini	81.72	Late Cretaceous	77.12–86.39
Andini + Cixiini	123.58	Early Cretaceous	121.4–125.77
Andini	89.47	Late Cretaceous	86.07–92.89
Cixiini	75.8	Late Cretaceous	71.83–79.82
*Andixius*	38.92	Eocene Paleogene	36.49–41.43
*Parandes* + *Andes*	79.19	Late Cretaceous	75.93–82.41
*Parandes*	44.9	Eocene Paleogene	42.42–47.38
*Andes*	55.71	Paleocene Paleogene	52.88–58.63

In Andini, the split of the clade *Andixius* was dated to the Eocene Paleogene (~38.92 Ma; 95% HPD: 36.49–41.43 Ma). The *Andes* + *Parandes* clade began radiating in the Late Cretaceous (~79.19 Ma; 95% HPD: 75.93–82.41 Ma), with *Andes* originating in the Paleocene Paleogene (~55.71 Ma) and *Parandes* separated at approximately the Eocene Paleogene (~44.9 Ma).

### 3.8 Ancestral State Reconstructions

Mesquite‐based reconstructions of the ScP + *R* and MP veins of forewing in the tribe Andini indicate that the ancestral condition is characterized by the veins arising from a common point on the basal cell (Figure 9). A single independent apomorphic transition is identified—from a common point to a common stalk arrangement—occurring specifically within the genus *Andixius*.

## 4 Discussion

Nine new Andini tribe cixiid mitogenomes were sequenced and analyzed in this work. A rearrangement of the *trnP*–*trnT* gene order was recovered in all nine assemblies. Such rearrangement is inconsistent with that observed in other cixiids but consistent with the gene order found in most delphacids (Ai et al. 2024). Earlier surveys of Fulgoroidea documented rearrangements—translocations or inversions—involving three PCGs (*nad4*, *nad4l*, *nad6*) and five tRNAs (*trnC*, *trnW*, *trnH*, *trnP*, and *trnT*) by comparison with the original arrangement (Song and Liang 2009; Zhang et al. 2013; Lv et al. 2015); none of these rearrangements are detected within the Andini genomes examined here.

The lengths of the nine Andini mitogenomes were tightly constrained (15,415–15,902 bp), contrasting with the wider span (14,949 bp in *Iolania perkinsi* –16,344 bp in Cixiidae sp.) reported for additional cixiid sequences used in this study (Chong et al. 2022; Wang, Meng, et al. 2024). Intergenic spacer variability and expansion/contraction of the AT‐rich control region account for most of the size differences (Wang, Li, et al. 2019; Gong et al. 2021b). All gene lengths are highly conserved and differ by < 20 bp among the Andini taxa. Four gene overlaps are invariant in this tribe: *trnW–trnC* (8 bp), *cox1–trnL2* (5 bp), *atp8–atp6* (11 bp), and *nad4–nad4l* (7 bp). Whereas the *trnW*/*trnC* and *atp8*/*atp6* overlaps are widespread in Cixiids and planthoppers (with variable lengths), the *nad4*/*nad4l* overlap is sporadically present (Xu et al. 2019; Gong et al. 2021a; Lv et al. 2021). The *cox1*/*trnL2* overlap is, to date, only observed in Andini.

We likewise examined AT and GC skews of the 13 PCGs across 16 cixiid mitogenomes. The L‐strand exhibits a negative AT skew and a positive GC skew, whereas the H‐strand shows an AT skew near zero and a consistently negative GC skew—patterns that align with previous findings in Fulgoroidea (Yu and Liang 2018; Xu et al. 2019; Lv et al. 2021). The nucleotide diversity and Ka/Ks values were obtained; among the 13 genes, *cox1* displays the lowest nucleotide diversity and smallest Ka/Ks ratio, consistent with strong purifying selection and its widespread use as a barcode locus (Demari‐Silva et al. 2015; Wang, Jiang, et al. 2024). All 22 tRNAs in Andini were predicted; the *trnS1* genes of *A. hemina*, *P. circinatus*, and *P. fuscus* lack the DHU stem, while the remaining 21 tRNA genes form typical cloverleaf secondary structures, a condition sporadically noted in other planthoppers (Wang, Huang, et al. 2019; Gong et al. 2021a, 2021b).

Phylogenetic inference (Figure 8) yielded congruent, fully supported topologies under both BI and ML criteria, with high support values. With *Borysthenes* used as an outgroup—representing a basal borystheninian lineage of Cixiidae—the Pentastirini tribe appears sister to the clade (Andini + Cixiini), echoing every previous reconstruction (Bucher et al. 2023; Ai et al. 2024; Luo et al. 2025) in agreement with Luo et al. (2025), contra Deng et al. (2025) whose conflicting result is likely due to insufficient taxon sampling.

Regarding the internal relationships of Andini, Deng et al. (2025) suggested a close affinity between Andini and Brixiini, although the monophyly of Andini cannot be reliably tested with only two taxa. Based on a broader taxon sampling, Luo et al. (2025) recover Andini as paraphyletic, with Brixiini nested within it, although in this last study, the identification of the Brixiini species still needs confirmation. We acknowledge that this study primarily focuses on the internal relationships within Andini and regret that we were unable to address the broader comparative issues here. However, these unresolved questions will be addressed in our future work. Within the Andini clade, the genus *Andixius* is placed at the base and is sister to a well‐supported monophyletic group comprising *Andes* and *Parandes* (BPP = 1; BP = 100).

**FIGURE 10:**
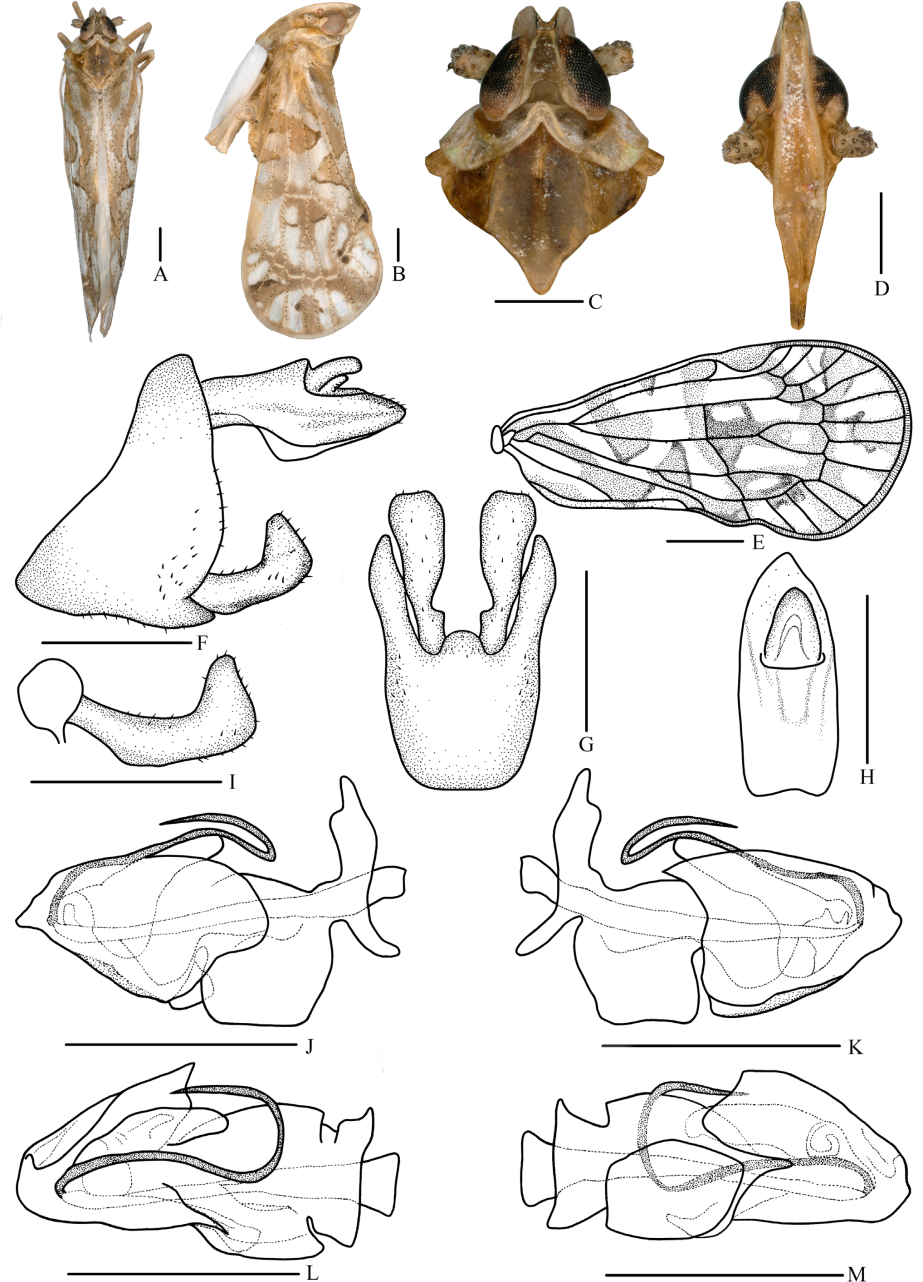
*Parandes hemina* (Fennah, 1978) comb. nov. (A) habitus, dorsal view, (B) habitus, lateral view, (C) head and thorax, dorsal view, (D) frons, ventral view, (E) forewing, (F) genitalia, lateral view, (G) pygofer and gonostyli, ventral view, (H) anal segment, dorsal view, (I) gonostyli, lateral view, (J) aedeagus, right side, (K) aedeagus, left side, (L) aedeagus, dorsal view, (M) aedeagus, ventral view. Scale bars: A–D, F–M = 0.5 mm, E = 1.0 mm.

In the genus *Andes*, the species *A. hemina* clusters with *Parandes*. *The species* was originally described by Fennah (1978) during his study of Cixiidae from Vietnam and was placed in the genus *Andes*. Although *Andes* and *Parandes* share similar external morphology, *Parandes* can be distinguished by the following features: head in profile with junction of vertex and frons slightly angular and produced; fore coxa produced and rounded on the outer edge of apical half; apex of periandrium with a long spinose process (Muir 1925; Wang, Zhi, Yang, Long, et al. 2023; Lv et al. 2025). The diagnostic illustration of *A. hemina* provided by Fennah (1978: Figure 13) clearly shows that the outer edge of the fore coxa on the basal segment is extended, smooth, and prominent—traits that correspond to those of the genus *Parandes*. Furthermore, the key morphological feature of *A. hemina* as illustrated in Figures 1 and 10 of this study is consistent with that of *Parandes*. Therefore, based on both phylogenetic evidence and morphological characters, we formally transfer the species *A. hemina* to the genus *Parandes* as *Parandes hemina* (Fennah, 1978) **comb. nov.**

Divergence‐time analyses (Figure 9) reveal that all Andini species experienced an accelerated radiation throughout the Cretaceous and Paleogene. This finding is consistent with previous statements (Bucher et al. 2023; Deng et al. 2025; Luo et al. 2025). The origin of Andini is estimated to be around 89.47 Ma, aligning with the results of Luo et al. (2025) and Deng et al. (2025). Besides, *Andixius* is dated 38.92 Ma, *Andes* diverged at 55.71 Ma in the Paleocene–Paleogene, and *Parandes* separated at approximately 44.9 Ma.

Finally, in our ancestral state reconstruction analysis (Figure 9), we examined the evolutionary transformation of the ScP + *R* and MP veins of forewing within the tribe Andini. The configuration in which ScP + *R* and MP arise from a common point on the basal cell appears to represent the ancestral condition. A derived condition, in which these veins form a short common stalk, is observed only within the *Andixius* clade, which emerged during the Eocene (~38 Ma).

In conclusion, our results improve understanding of mitochondrial genome evolution in Cixiidae and particularly in the Andini tribe and offer new perspectives on the tribe's phylogenetic relationships, divergence history, and the evolution of morphological characters.

## 5 Taxonomic Decisions

*Parandes* Muir, 1925.

*Parandes* Muir, 1925: 511; Wang, Zhi, Yang, Long, et al. 2023: 112.

*Type species*: *Parandes simplus* Muir, 1925, original designation.

*Diagnosis*: Head in profile with the junction of vertex and frons slightly angular and produced. Frons narrow and long. ScP + *R* and MP veins of forewing arising separately from a common point on the basal cell. Fore coxa produced and rounded on the outer edge of the apical half. Hind tibiae without or with several very small lateral spines. Apex of periandrium with a long spinose process; endosoma simple, without process.

*Distribution*: China, Indonesia, Malaysia, Vietnam.

Checklist and distributions of species of *Parandes* Muir, 1925.

*P. circinatus* Wang & Chen, 2023; China (Yunnan).

*P. elongatus* Lv & Chen, 2025; China (Yunnan).

*P. fuscus* Wang & Chen, 2023; China (Yunnan).

*P. guangxiensis* Lv & Chen, 2025; China (Guangxi).

*P. hamatus* Lv & Chen, 2025; China (Yunnan).

*P. hemina* (Fennah, 1978) comb. nov.; China (Yunnan, Guangxi), Malaysia (Kuala Lumpur, Kedah), Vietnam (Ninh Bình).

*P. simplus* Muir, 1925; Indonesia (West Borneo).

*Parandes hemina* (Fennah, 1978) comb. nov.

Figure 10.

*Andes hemina* Fennah, 1978: 209.

*Material examined*: CHINA • 20♂♂; Guangxi Zhuang Autonomous Region, Mulun National Nature Reserve; 25°15ʹN, 108°07ʹE; sweeping, 28 July 2019; Yong‐Jin Sui, Zhi‐Cheng Zhou, Xiao‐Ya Wang, Jing Wang leg.; IEGU.

*Redescription*: *Measurements*. Total Length: Male 5.81–6.44 mm (*N* = 20).

*Coloration*: General color light yellowish brown (Figure 10A,B). Vertex brown. Eyes blackish brown. Ocelli light yellow with red. Frons yellowish brown, lateral side of head with a triangular brown spot anterior to the eyes. Clypeus yellowish brown. Antennae light grayish brown. Pronotum with part behind eyes brown, the rest grayish brown. Mesonotum dark brown. Tegula yellowish brown. Forewings semi‐translucent, light yellowish brown, with many variform grayish‐white stripes and markings, a looped band from basal costal margin obliquely across to CuP, recurved to 1/3 costal margin, stigma dull yellow, veins grayish‐white, tubercles brown, as shown in Figure 10E.

*Head and thorax*: Vertex (Figure 10A,C) 1.21 times as long as wide, width at apex narrower than at base (1:2.21), anterior margin nearly straight, posterior margin U‐shaped recessed, lateral carina developed, median carina absent. Frons (Figure 10D) longer in middle line than wide at widest portion (about 3.96:1), widest at nearly apex, lateral carina developed, apex of median carina raised. Clypeus (Figure 10D) with distinct median carina. Pronotum (Figure 10A,C) shorter than vertex in midline (1:2.16), posterior margin recessed. Mesonotum (Figure 10A,C) longer than 1.72 times pronotum and vertex combined. Forewings (Figure 10E) 2.07 times as long as wide, with 12 apical cells and seven subapical cells, RP 3 branches, MP with 5 terminals: MP_11_, MP_12_, MP_2_, MP_3_, and MP_4_, fork MP_1_ + MP_2_ basad of fork MP_3_ + MP_4_. Hind tibia with five lateral spines.

*Male genitalia*: Pygofer (Figure 10F,G) ventral margin distinctly longer than dorsal margin in lateral view, posterior margin convex at middle, lateral lobes arcuate and extended caudally; in ventral view symmetrical, medioventral process nearly semicircular. Anal segment (Figure 10F,H) asymmetrical, in lateral view flat tubular, dorsal margin straight, ventral margin curved slightly; in dorsal view, 2.46 times as long as wide; anal style big, triangular, not extending beyond anal segment. Gonostyli (Figure 10G,I) in lateral view L‐shaped, curved dorsally near the middle; in ventral view lateral margins curved, widens toward the end, baseball‐shaped, basal 1/3 with emarginations. Aedeagus (Figure 10J–M) with a spinose process. Periandrium with a lamellar process at apical half, ventral side with a nearly rectangular lamellar process at basal half; apical part with a slender spinous process, curved ventrally, apical 1/4 recurved, directed caudad. Endosoma slightly sclerotized, without process.

*Distribution*: China (Yunnan, Guangxi), Malaysia (Kuala Lumpur, Kedah), Vietnam (Ninh Bình).

## Author Contributions

**Sha‐Sha Lv:** data curation (equal), formal analysis (equal), methodology (equal), writing – original draft (equal), writing – review and editing (equal). **Thierry Bourgoin:** conceptualization (equal), writing – review and editing (equal). **Xiao‐Ya Wang:** conceptualization (equal), methodology (equal). **Lin Yang:** conceptualization (equal), resources (equal), writing – review and editing (equal). **Yu‐Bo Zhang:** methodology (equal), software (equal), writing – review and editing (equal). **Xiang‐Sheng Chen:** funding acquisition (lead), project administration (lead), supervision (lead), writing – review and editing (equal).

## Conflicts of Interest

The authors declare no conflicts of interest.

## Supporting information


**Figure S1:** Predicted secondary structures of the 22 tRNAs of *Andes bifidus* mitogenome.
**Figure S2:** Predicted secondary structures of the 22 tRNAs of *Andes furcutus* mitogenome.
**Figure S3:** Predicted secondary structures of the 22 tRNAs of *Andes hemina* mitogenome.
**Figure S4:** Predicted secondary structures of the 22 tRNAs of *Andes latanalus* mitogenome.
**Figure S5:** Predicted secondary structures of the 22 tRNAs of *Andes pallidus* mitogenome.
**Figure S6:** Predicted secondary structures of the 22 tRNAs of *Andixius cultratus* mitogenome.
**Figure S7:** Predicted secondary structures of the 22 tRNAs of *Andixius truncatus* mitogenome.
**Figure S8:** Predicted secondary structures of the 22 tRNAs of *Parandes circinatus* mitogenome.
**Figure S9:** Predicted secondary structures of the 22 tRNAs of *Parandes fuscus* mitogenome.

**Table S1:** The information of nine Andini species in this study.
**Table S2:** Best partitioning schemes and models for phylogenetic analysis based on 13 PCGs.
**Table S3:** Organization of the nine Andini species mitochondrial genomes.
**Table S4:** Nucleotide composition of tribe Andini planthopper mitochondrial genome.
**Table S5:** Start and stop codons in the mitochondrial genomes of tribe Andini.
**Table S6:** Codon numbers and RSCU in mitochondrial PCGs of Andini species.
**Table S7:** AT and GC skews of 13 PCGs across 16 Cixiidae mitogenomes.
**Table S8:** The nucleotide diversity values of 13 PCGs in Andini.
**Table S9:** The Ka, Ks, and Ka/Ks values for the 13 PCGs of the nine Andini species.

## Data Availability

The mitochondrial genome of nine Andini species in this study is available from the NCBI GenBank under the accession numbers listed in Table 1 (2. Materials and Methods).
