# Supplementary material for: Comparative Mitogenomics of Andini (Hemiptera: Cixiidae) Reveals Rapid Radiation, Clarifies Relationships, and Supports Taxonomic Revision Within the Tribe
Source: Ecol Evol. 2025 Oct 20;15(10):e72321. doi: 10.1002/ece3.72321 (PMC12537841; doi:10.1002/ece3.72321)
Supplement: Supplementary file 1 — Figure S1: Predicted secondary structures of the 22 tRNAs of Andes bifidus mitogenome. Figure S2: Predicted secondary structures of the 22 tRNAs of Andes furcutus mitogenome. Figure S3: Predicted secondary structures of the 22 tRNAs of Andes hemina mitogenome. Figure S4: Predicted secondary structures of the 22 tRNAs of Andes latanalus mitogenome. Figure S5: Predicted secondary structures of the 22 tRNAs of Andes pallidus mitogenome. Figure S6: Predicted secondary structures of the 22 tRNAs of Andixius cultratus mitogenome. Figure S7: Predicted secondary structures of the 22 tRNAs of Andixius truncatus mitogenome. Figure S8: Predicted secondary structures of the 22 tRNAs of Parandes circinatus mitogenome. Figure S9: Predicted secondary structures of the 22 tRNAs of Parandes fuscus mitogenome. [file ECE3-15-e72321-s002.docx]

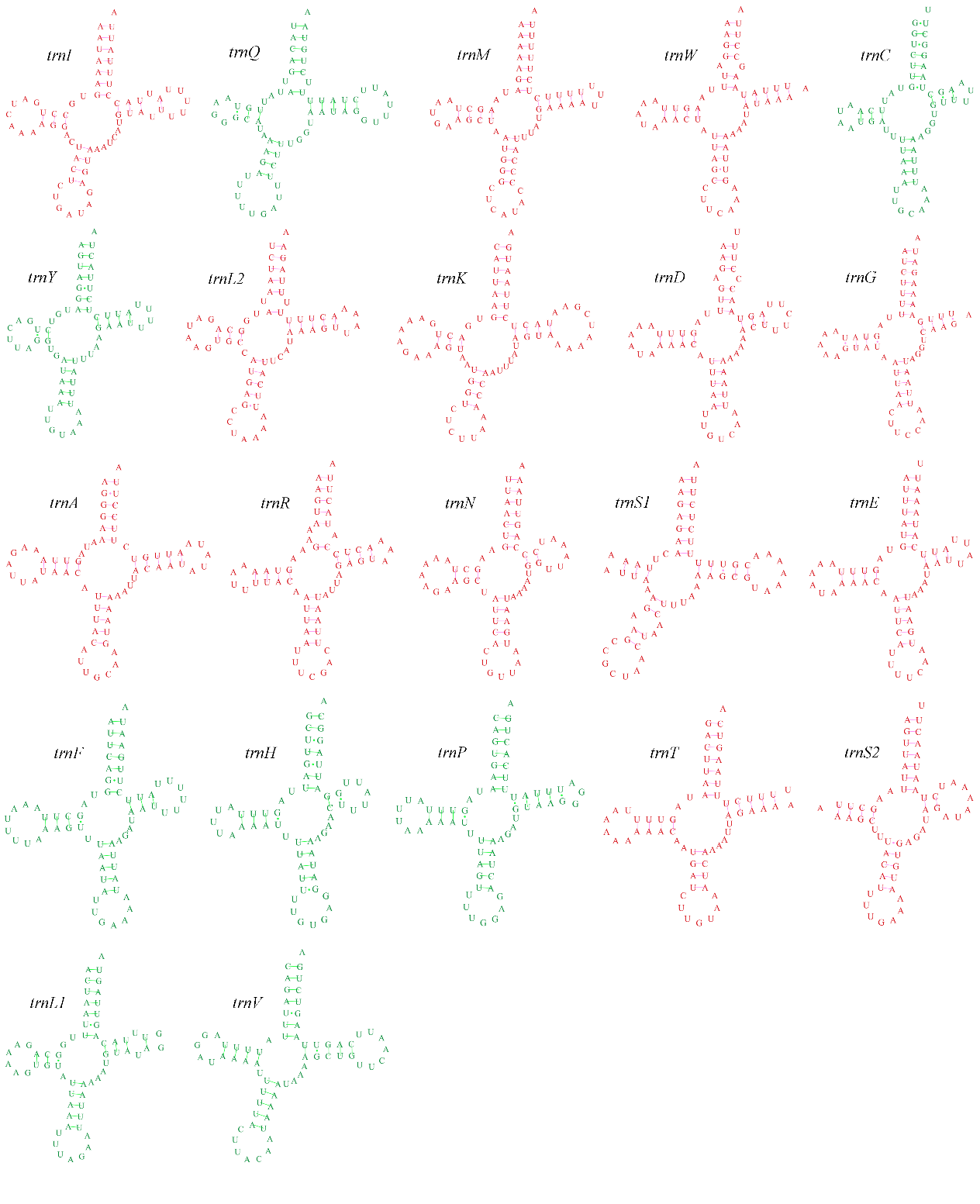


FIGURE S1. Predicted secondary structures of the 22 tRNAs of *Andes bifidus* mitogenome.


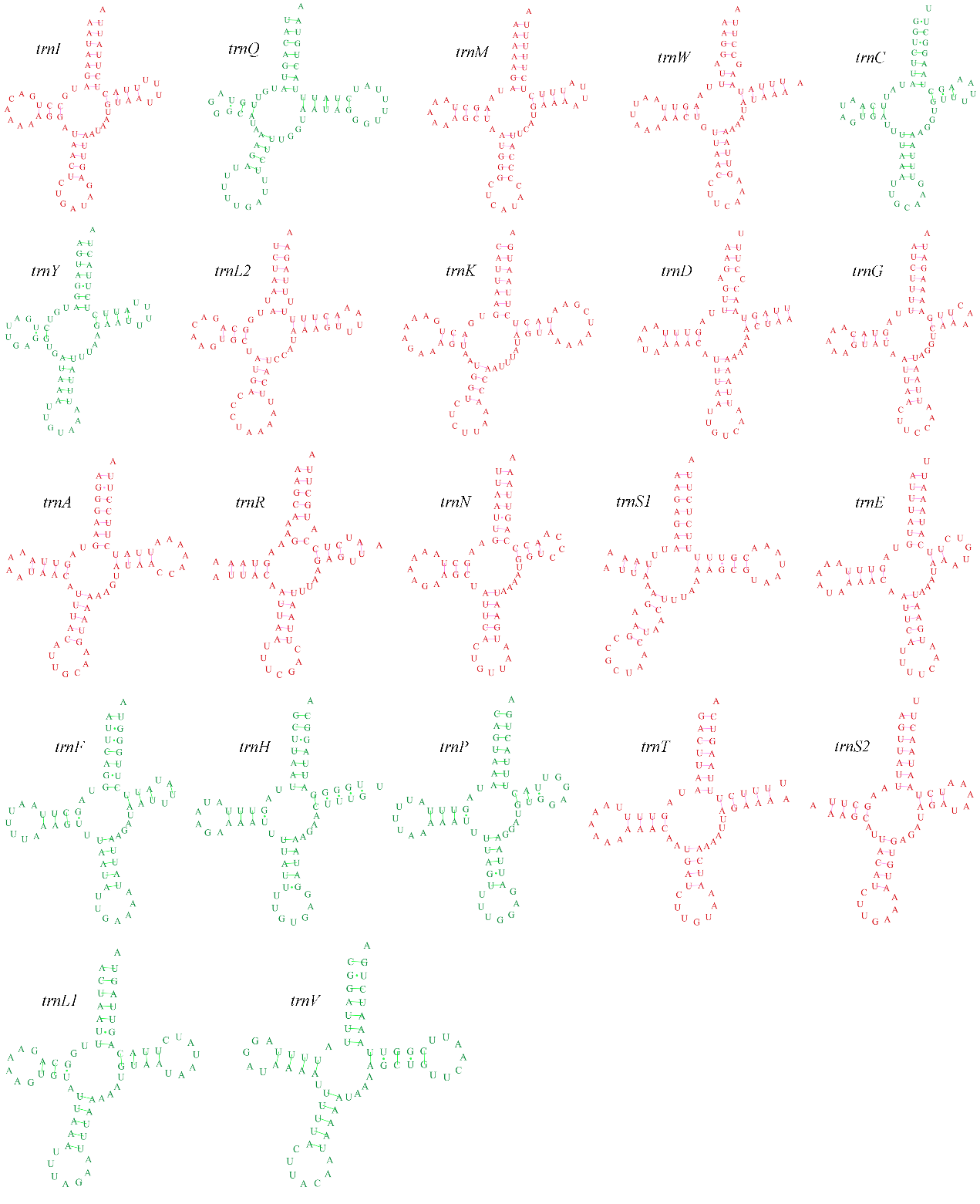


FIGURE S2. Predicted secondary structures of the 22 tRNAs of *Andes furcutus* mitogenome.


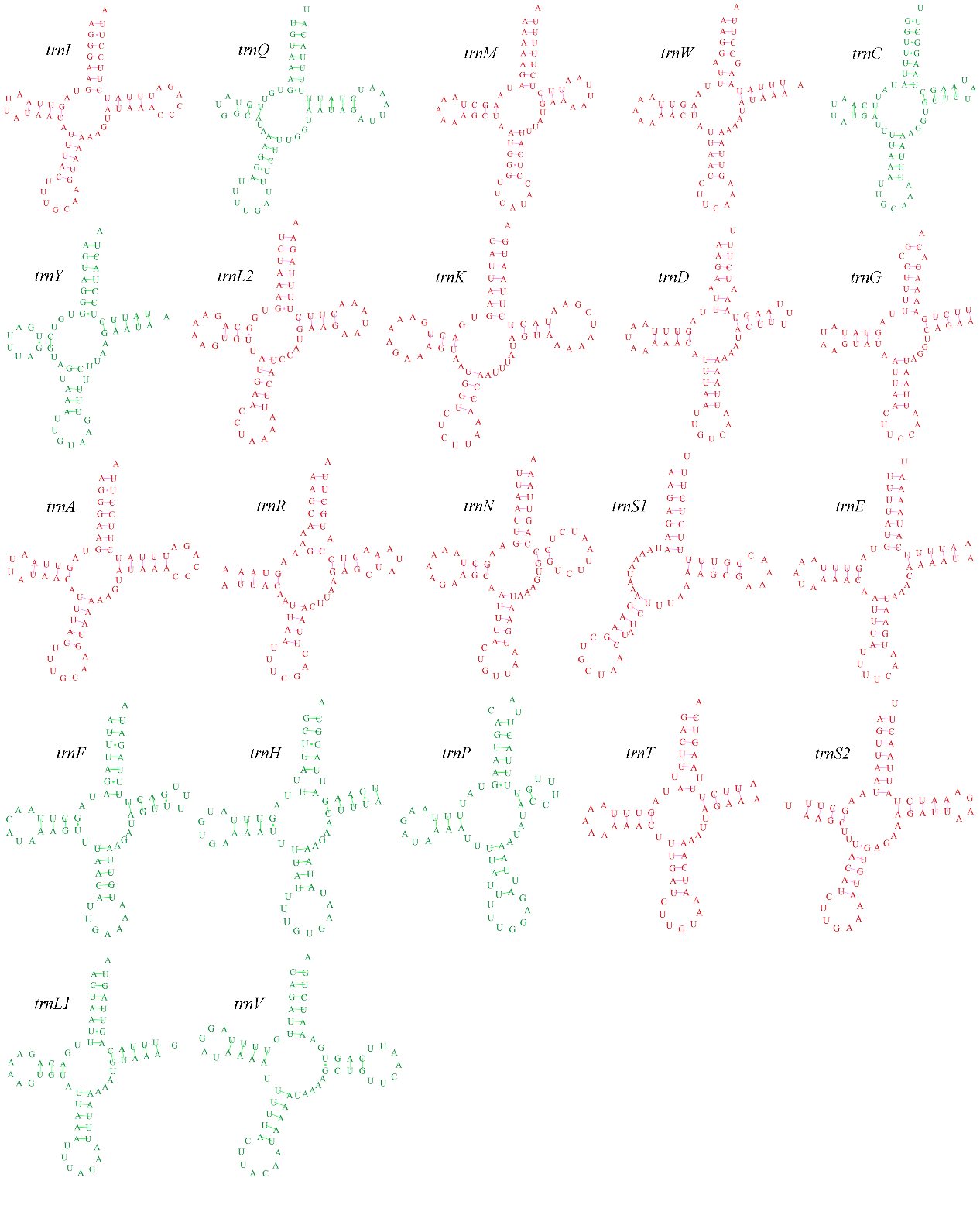


FIGURE S3. Predicted secondary structures of the 22 tRNAs of *Andes hemina* mitogenome.


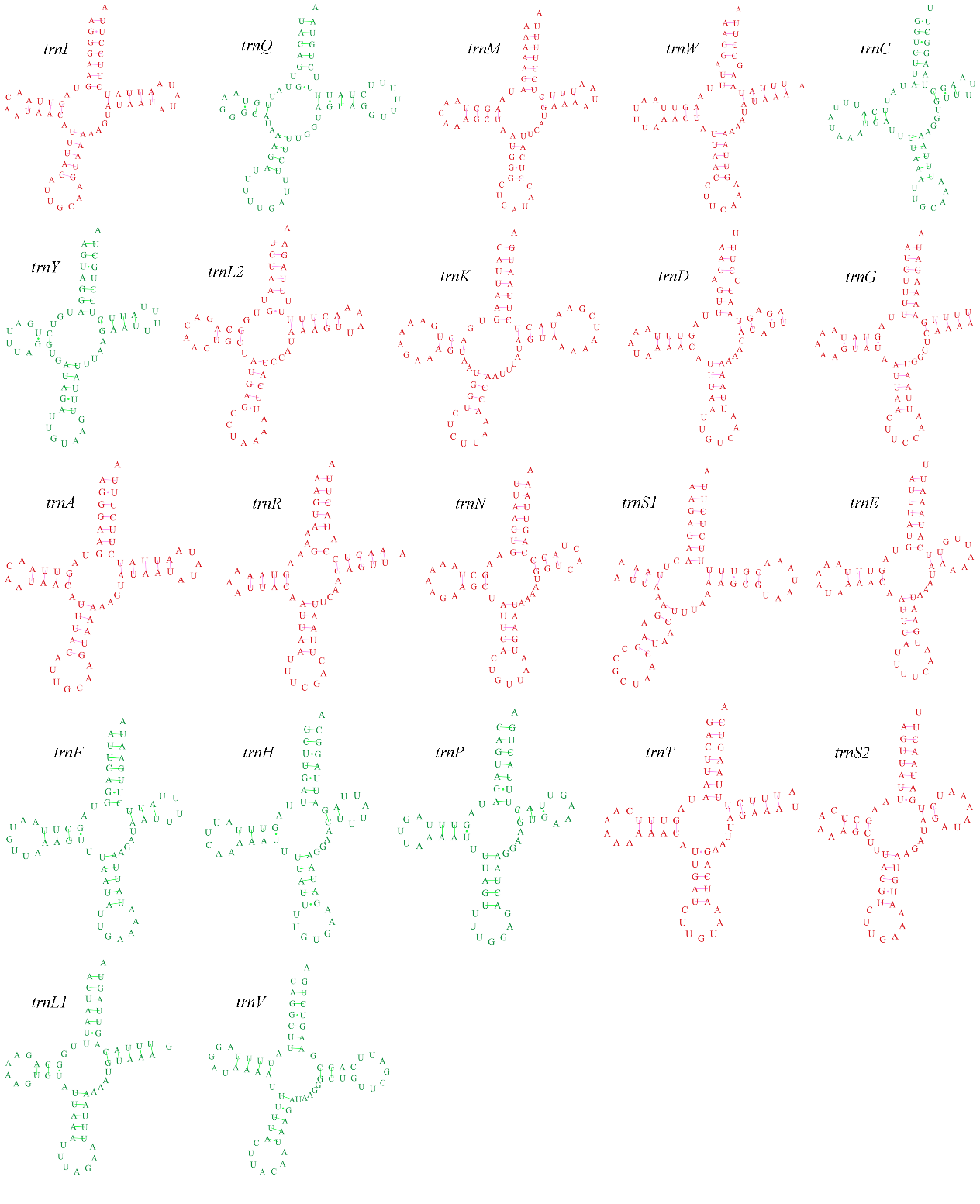


FIGURE S4. Predicted secondary structures of the 22 tRNAs of *Andes latanalus* mitogenome.


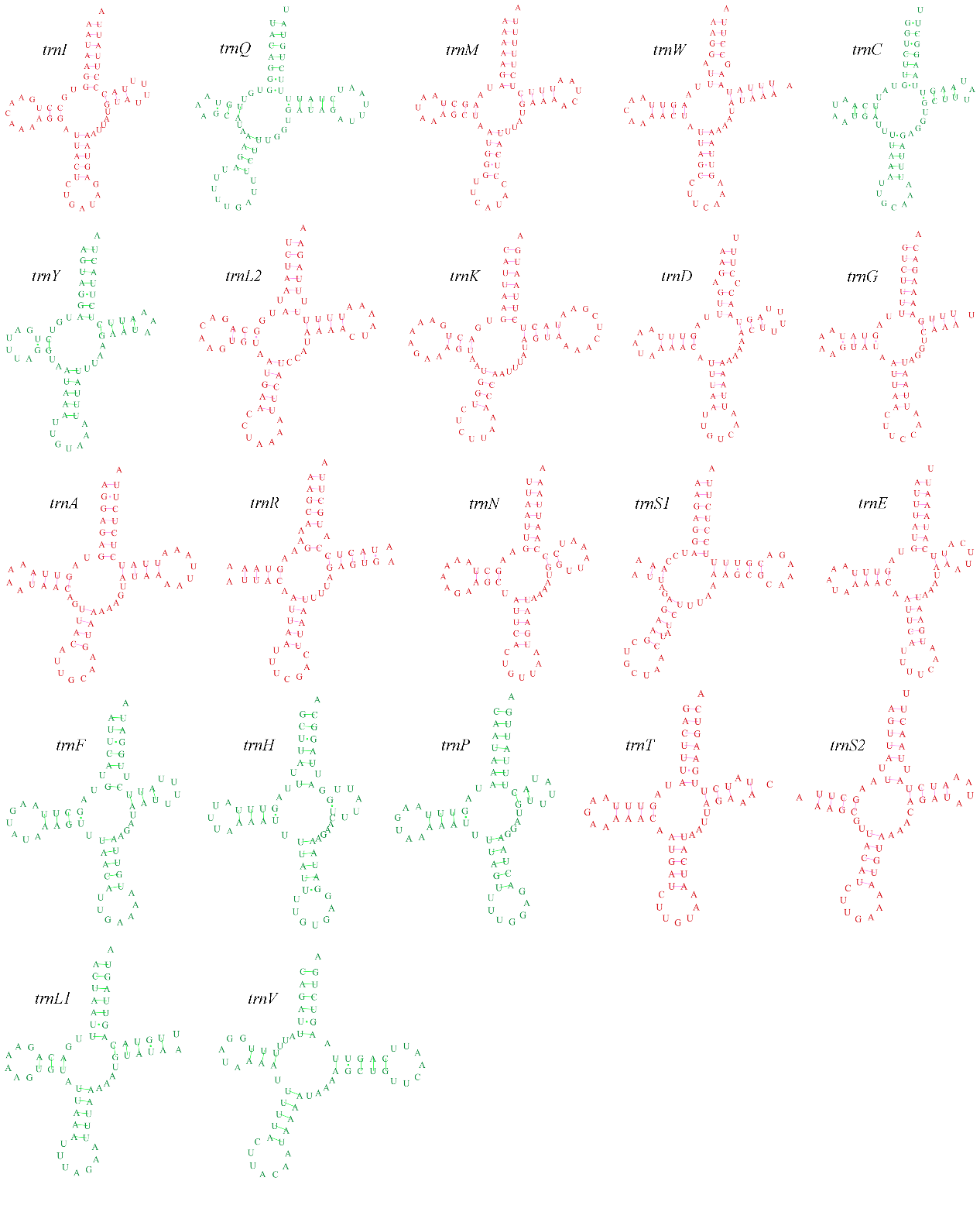


FIGURE S5. Predicted secondary structures of the 22 tRNAs of *Andes pallidus* mitogenome.


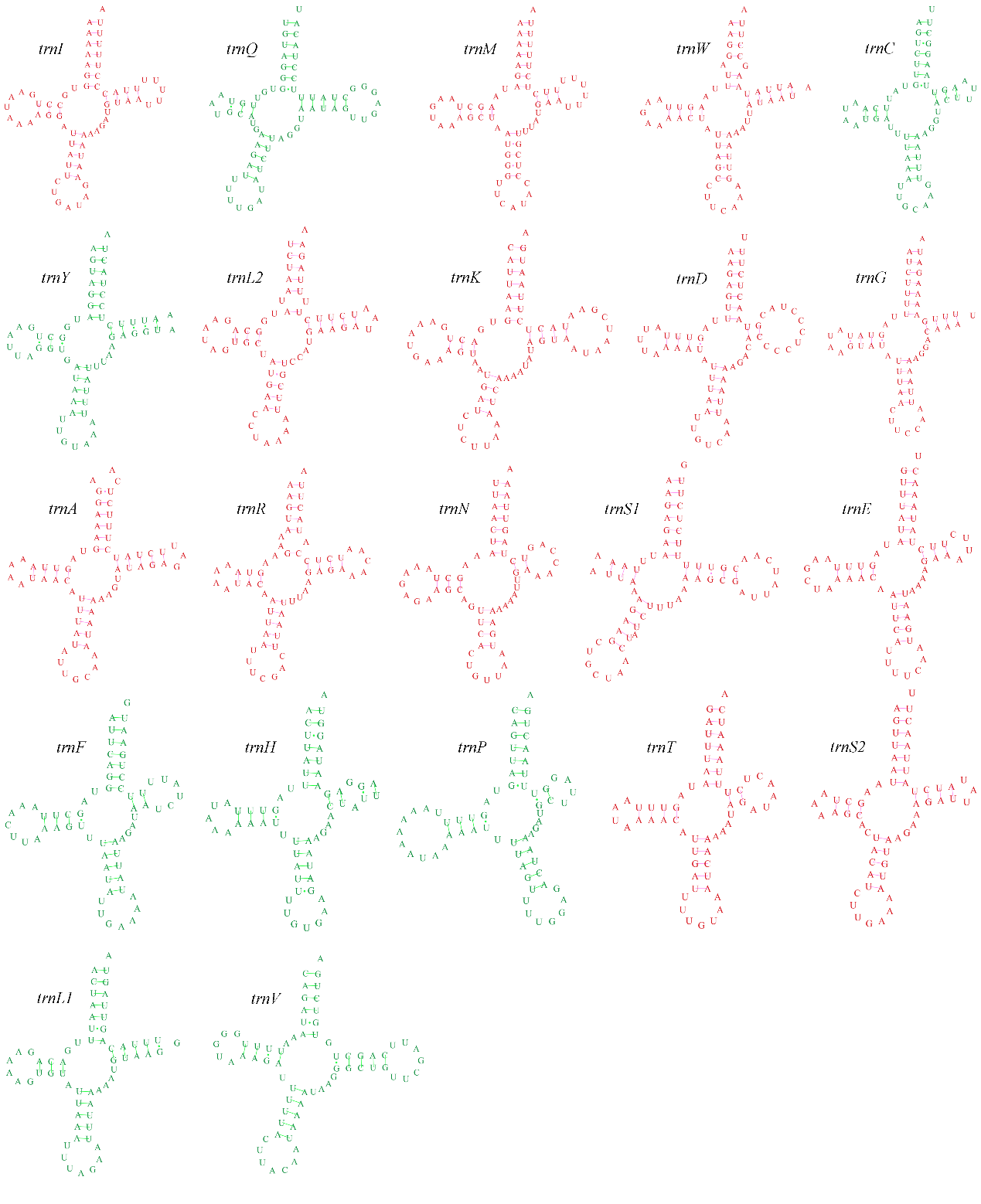


FIGURE S6. Predicted secondary structures of the 22 tRNAs of *Andixius cultratus* mitogenome.


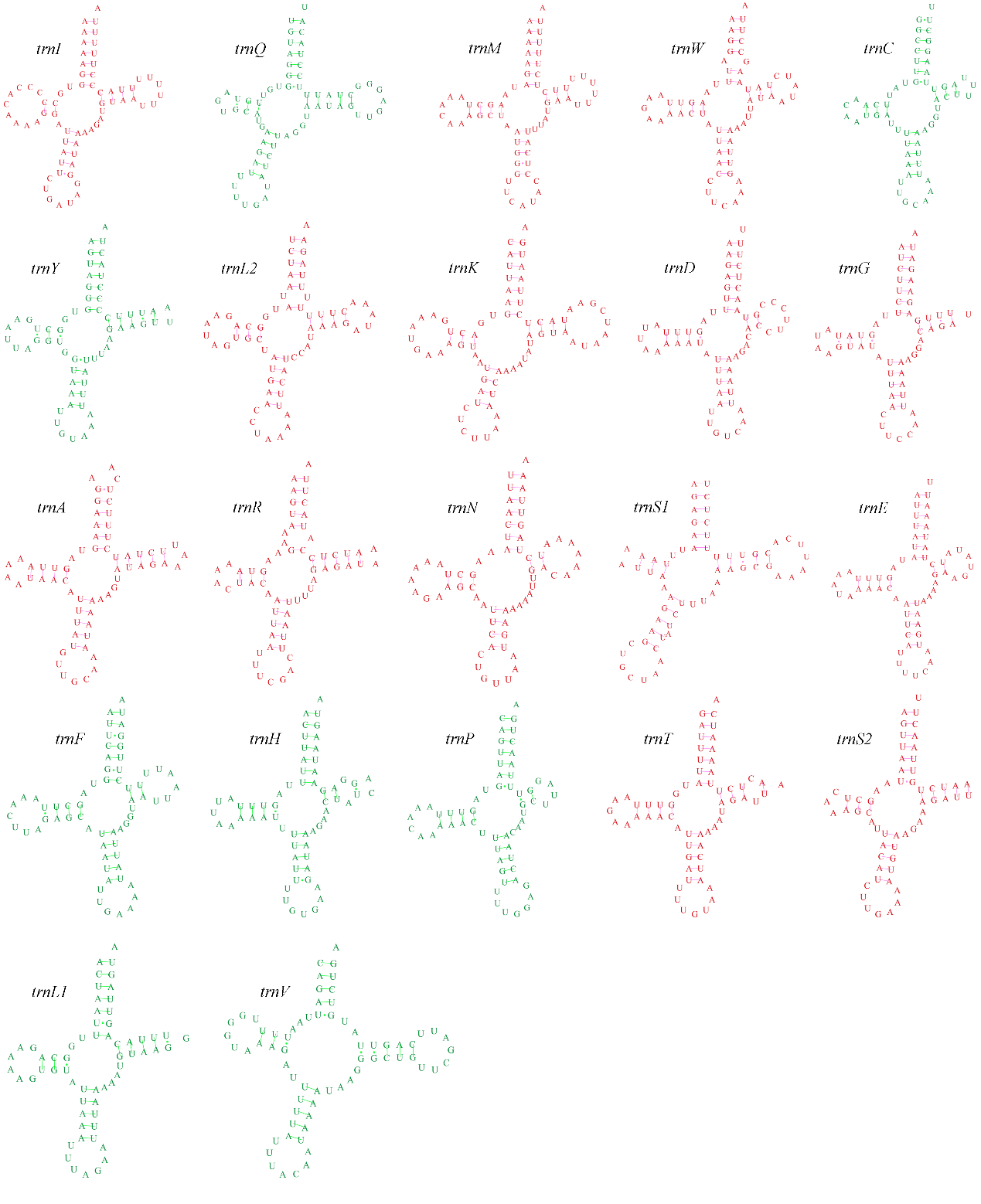


FIGURE S7. Predicted secondary structures of the 22 tRNAs of *Andixius truncatus* mitogenome.


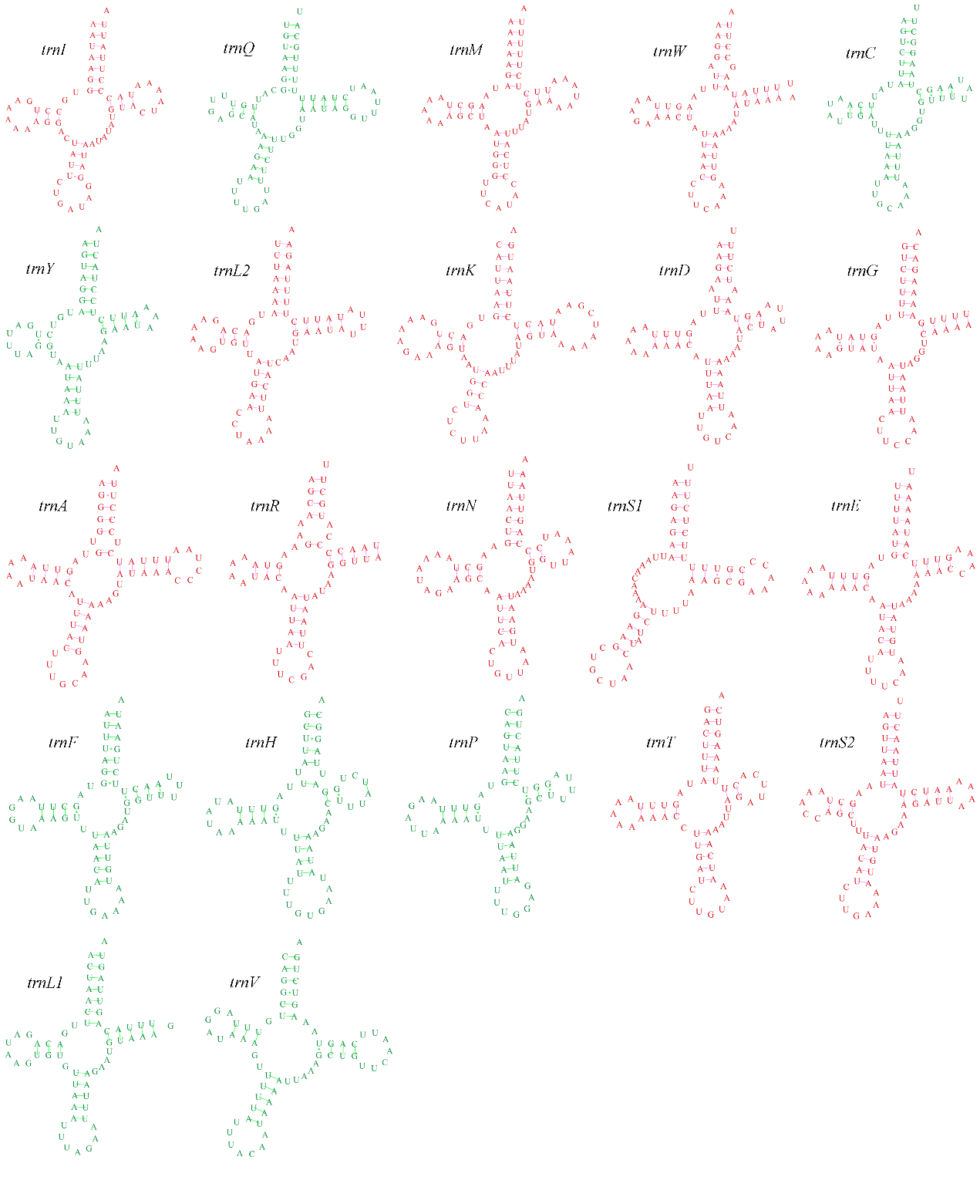


FIGURE S8. Predicted secondary structures of the 22 tRNAs of *Parandes circinatus* mitogenome.


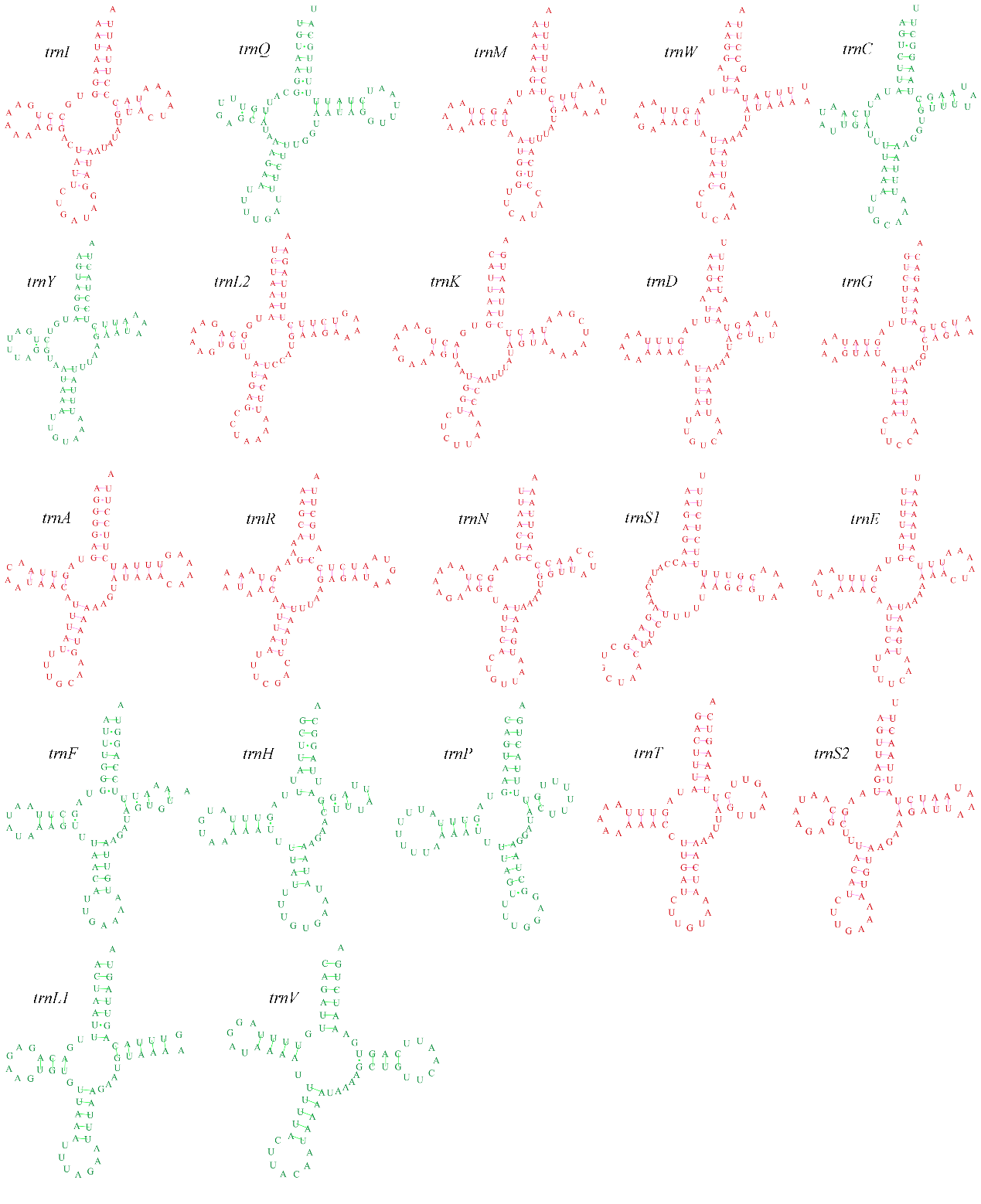


FIGURE S9. Predicted secondary structures of the 22 tRNAs of *Parandes fuscus* mitogenome.
