# Supplementary material for: Comparative Mitogenomics of Andini (Hemiptera: Cixiidae) Reveals Rapid Radiation, Clarifies Relationships, and Supports Taxonomic Revision Within the Tribe
Source: Ecol Evol. 2025 Oct 20;15(10):e72321. doi: 10.1002/ece3.72321 (PMC12537841; doi:10.1002/ece3.72321)
Supplement: Supplementary file 2 — Table S1: The information of nine Andini species in this study. Table S2: Best partitioning schemes and models for phylogenetic analysis based on 13 PCGs. Table S3: Organization of the nine Andini species mitochondrial genomes. Table S4: Nucleotide composition of tribe Andini planthopper mitochondrial genome. Table S5: Start and stop codons in the mitochondrial genomes of tribe Andini. Table S6: Codon numbers and RSCU in mitochondrial PCGs of Andini species. Table S7: AT and GC skews of 13 PCGs across 16 Cixiidae mitogenomes. Table S8: The nucleotide diversity values of 13 PCGs in Andini. Table S9: The Ka, Ks, and Ka/Ks values for the 13 PCGs of the nine Andini species. [file ECE3-15-e72321-s001.docx]

Table S1. The information of nine Andini species in this study.

| Organism | Geographical locality | Collector | Date |
| --- | --- | --- | --- |
| *Andes bifidus* | Pingbian, Yunnan, China | Y.-J. Sui | 18-Aug-17 |
| *Andes furcutus* | Jiangkou, Guizhou, China | F.-E. Li | 4-Jun-20 |
| *Andes hemina* | Mulun, Guangxi, China | X.-Y. Wang | 28-Jul-19 |
| *Andes latanalus* | Mulun, Guangxi, China | Z.-C. Zhou | 29-Jul-19 |
| *Andes pallidus* | Wangmo, Guizhou, China | S.-S. Lv | 14-Aug-20 |
| *Andixius cultratus* | Chebaling, Guangdong, China | S.-S. Lv | 14-Jun-23 |
| *Andixius truncatus* | Longsheng, Guangxi, China | X.-Y. Wang | 18-Jul-20 |
| *Parandes circinatus* | Mengla, Yunnan, China | F.-E. Li | 13-Jun-19 |
| *Parandes fuscus* | Mengla, Yunnan, China | F.-E. Li | 13-Jun-19 |

Table S2. Best partitioning schemes and models for phylogenetic analysis based on 13 PCGs.

| Dataset | Partitioning scheme | Models |
| --- | --- | --- |
| PCGs | S1: *cox2*,*cytb*,*cox3*,*nad3*,*atp6* | GTR+I+G |
|  | S2: *nad6*,*atp8*,*nad2* | TVM+I+G |
|  | S3: *cox1* | GTR+I+G |
|  | S4: *nad1*,*nad4l*,*nad5*,*nad4* | TIM+I+G |

Table S3. Organization of the nine Andini species mitochondrial genomes.

|  | *Andes bifidus/Andes furcutus/Andes hemina/Andes latanalus/Andes pallidus/Andixius cultratus/Andixius truncatus/Parandes circinatus/Parandes fuscus* | | |
| --- | --- | --- | --- |
| Name | Size | Intergenic Nucleotide | Direction |
| *trnI* | 68/65/64/65/63/66/67/64/64 | 0/0/0/0/0/0/0/0/0 | H |
| *trnQ* | 68/68/67/68/67/67/67/68/68 | 7/2/-1/2/-1/-1/-1/1/8 | L |
| *trnM* | 65/64/64/64/65/65/65/64/65 | -1/-1/-1/-1/-1/-1/-1/-1/0 | H |
| *nad2* | 960/960/960/960/960/960/960/960/960 | 0/0/0/0/0/0/0/0/0 | H |
| *trnW* | 63/65/63/64/63/63/63/64/63 | -1/-1/-1/-1/-1/-2/-2/-1/-1 | H |
| *trnC* | 60/61/62/64/62/60/60/62/63 | -8/-8/-8/-8/-8/-8/-8/-8/-8 | L |
| *trnY* | 63/63/62/63/62/63/63/62/62 | 9/4/7/4/0/0/0/1/1 | L |
| *cox1* | 1503/1503/1503/1503/1503/1503/1503/1503/1503 | 34/34/34/34/34/34/34/34/34 | H |
| *trnL2* | 65/65/65/65/65/64/64/65/65 | -5/-5/-5/-5/-5/-5/-5/-5/-5 | H |
| *cox2* | 670/670/670/670/670/667/667/673/670 | 1/1/1/1/1/1/1/1/1 | H |
| *trnK* | 71/71/71/71/71/71/71/71/71 | 1/1/1/1/1/1/1/1/1 | H |
| *trnD* | 64/62/63/62/62/66/62/62/63 | 1/0/0/2/0/0/0/0/0 | H |
| *atp8* | 102/102/102/102/102/102/102/102/102 | 0/0/0/0/0/0/0/0/0 | H |
| *atp6* | 655/655/655/655/655/655/655/655/655 | -11/-11/-11/-11/-11/-11/-11/-11/-11 | H |
| *cox3* | 783/783/783/783/783/783/783/783/783 | 0/0/0/0/0/0/0/0/0 | H |
| *trnG* | 61/61/61/62/60/60/60/61/61 | 5/3/0/5/0/0/0/0/0 | H |
| *nad3* | 351/351/351/351/351/351/351/351/351 | 0/0/0/0/0/0/0/0/0 | H |
| *trnA* | 69/66/67/66/66/64/64/67/67 | -2/-2/4/-2/-2/-2/-2/0/2 | H |
| *trnR* | 63/61/63/62/62/63/62/59/65 | 0/4/1/2/-1/-1/-1/0/0 | H |
| *trnN* | 63/63/66/63/63/63/64/64/65 | -3/-3/-3/-3/-3/-3/-3/-2/-3 | H |
| *trnS1* | 65/65/60/65/64/66/62/61/60 | -1/-1/-1/-1/-1/-1/0/-1/-1 | H |
| *trnE* | 63/62/64/63/61/63/62/64/64 | -1/-1/-1/-1/-1/-1/1/-1/-1 | H |
| *trnF* | 67/64/64/64/64/64/64/64/64 | 0/2/0/0/1/0/0/7/7 | L |
| *nad5* | 1684/1690/1687/1684/1684/1687/1687/1687/1684 | 0/1/0/0/1/0/0/0/0 | L |
| *trnH* | 61/64/63/62/61/62/62/63/63 | 0/0/0/0/0/0/0/0/0 | L |
| *nad4* | 1315/1315/1312/1315/1315/1317/1317/1312/1315 | 1/1/3/1/1/-1/3/3/0 | L |
| *nad4l* | 276/276/276/276/276/276/276/276/276 | -7/-7/-7/-7/-7/-7/-7/-7/-7 | L |
| *trnP* | 66/65/61/63/61/65/61/64/66 | 69/73/69/70/75/71/72/0/72 | L |
| *trnT* | 65/66/62/65/63/61/63/61/63 | -1/-1/4/-1/-1/6/7/9/9 | H |
| *nad6* | 496/496/496/496/496/493/493/496/496 | 0/0/3/0/1/1/1/2/5 | H |
| *cytb* | 1122/1122/1122/1122/1122/1122/1122/1122/1122 | 0/0/0/0/0/0/0/0/0 | H |
| *trnS2* | 60/60/63/61/61/60/60/65/65 | -2/-2/-2/2/-2/-2/-2/-2/-2 | H |
| *nad1* | 916/916/916/916/916/916/916/916/916 | 15/15/15/15/15/15/15/15/15 | L |
| *trnL1* | 63/66/62/62/63/62/62/62/63 | 1/1/1/1/1/1/1/1/1 | L |
| *rrnL* | 1206/1208/1205/1210/1203/1199/1193/1197/1211 | 0/0/0/0/0/0/0/0/0 | L |
| *trnV* | 68/68/67/68/67/68/68/66/67 | 0/0/0/0/0/0/0/0/0 | L |
| *rrnS* | 738/737/738/737/750/719/738/743/720 | 0/0/0/0/0/0/0/0/0 | L |
| Control region | 1484/1379/1552/1426/1310/1175/1650/1230/1459 | 0/0/0/0/0/0/0/0/0 | H |
| Full genomes | 15783/15677/15834/15717/15578/15415/15902/15445/15757 |  |  |

Table S4. Nucleotide composition of tribe Andini planthopper mitochondrial genome.

| *Andes bifidus* | | | | | | | | | |
| --- | --- | --- | --- | --- | --- | --- | --- | --- | --- |
| Regions | Size (bp) | T(U)% | C% | A% | G% | AT% | GC% | AT skew | GC skew |
| Full genomes | 15783 | 30.2 | 16.7 | 44.6 | 8.5 | 74.8 | 25.2 | 0.1930 | -0.3250 |
| PCGS | 10827 | 42.9 | 14 | 30.8 | 12.3 | 73.7 | 26.3 | -0.1642 | -0.0646 |
| tRNAs | 1421 | 37.4 | 9.6 | 39.9 | 13.1 | 77.3 | 22.7 | 0.0323 | 0.1542 |
| rRNAs | 1944 | 46.2 | 7.4 | 27.7 | 18.7 | 73.9 | 26.1 | -0.2503 | 0.4330 |
| Control region | 1484 | 35.2 | 13.1 | 44.6 | 7.1 | 79.8 | 20.2 | 0.1178 | -0.2970 |
|  |  |  |  |  |  |  |  |  |  |
| *Andes furcutus* | | | | | | | | | |
| Regions | Size (bp) | T(U)% | C% | A% | G% | AT% | GC% | AT skew | GC skew |
| Full genomes | 15677 | 30.2 | 17.5 | 43.3 | 8.9 | 73.5 | 26.4 | 0.1780 | -0.3258 |
| PCGS | 10833 | 42.6 | 14.8 | 30.1 | 12.5 | 72.7 | 27.3 | -0.1719 | -0.0843 |
| tRNAs | 1415 | 35.9 | 10.1 | 40.3 | 13.7 | 76.2 | 23.8 | 0.0577 | 0.1513 |
| rRNAs | 1945 | 40.8 | 9.3 | 32 | 17.9 | 72.8 | 27.2 | -0.1209 | 0.3162 |
| Control region | 1379 | 38.1 | 14.4 | 39.1 | 8.4 | 77.2 | 22.8 | 0.0130 | -0.2632 |
|  |  |  |  |  |  |  |  |  |  |
| *Andes hemina* |  |  |  |  |  |  |  |  |  |
| Regions | Size (bp) | T(U)% | C% | A% | G% | AT% | GC% | AT skew | GC skew |
| Full genomes | 15834 | 29.9 | 16.1 | 44.9 | 9.1 | 74.8 | 25.2 | 0.2005 | -0.2778 |
| PCGS | 10827 | 42.7 | 14.3 | 30.3 | 12.7 | 73 | 27 | -0.1699 | -0.0593 |
| tRNAs | 1404 | 36.1 | 10 | 40.5 | 13.3 | 76.6 | 23.3 | 0.0574 | 0.1416 |
| rRNAs | 1943 | 47.4 | 7.7 | 27.9 | 16.9 | 75.3 | 24.6 | -0.2590 | 0.3740 |
| Control region | 1552 | 37.1 | 10.2 | 46.9 | 5.7 | 84 | 16 | 0.1167 | -0.2813 |
|  |  |  |  |  |  |  |  |  |  |
| *Andes latanalus* |  |  |  |  |  |  |  |  |  |
| Regions | Size (bp) | T(U)% | C% | A% | G% | AT% | GC% | AT skew | GC skew |
| Full genomes | 15717 | 30.2 | 16.8 | 44.6 | 8.3 | 74.8 | 25.1 | 0.1925 | -0.3387 |
| PCGS | 10827 | 42.5 | 14.4 | 30.5 | 12.6 | 73 | 27 | -0.1644 | -0.0667 |
| tRNAs | 1412 | 36 | 10.5 | 39.2 | 14.3 | 75.2 | 24.8 | 0.0426 | 0.1532 |
| rRNAs | 1947 | 46.7 | 7.6 | 30.1 | 15.5 | 76.8 | 23.1 | -0.2162 | 0.3420 |
| Control region | 1426 | 42.1 | 9.7 | 43.5 | 4.8 | 85.6 | 14.4 | 0.0164 | -0.3403 |
|  |  |  |  |  |  |  |  |  |  |
| *Andes pallidus* |  |  |  |  |  |  |  |  |  |
| Regions | Size (bp) | T(U)% | C% | A% | G% | AT% | GC% | AT skew | GC skew |
| Full genomes | 15578 | 31.6 | 16 | 44.4 | 8 | 76 | 24 | 0.1684 | -0.3333 |
| PCGS | 10827 | 43.5 | 13.7 | 31 | 11.7 | 74.5 | 25.4 | -0.1678 | -0.0787 |
| tRNAs | 1396 | 36.3 | 10.1 | 40.2 | 13.4 | 76.5 | 23.5 | 0.0510 | 0.1404 |
| rRNAs | 1953 | 45.7 | 7.3 | 30.7 | 16.3 | 76.4 | 23.6 | -0.1963 | 0.3814 |
| Control region | 1310 | 42.9 | 9.1 | 43.3 | 4.7 | 86.2 | 13.8 | 0.0046 | -0.3188 |
|  |  |  |  |  |  |  |  |  |  |
| *Andixius cultratus* |  |  |  |  |  |  |  |  |  |
| Regions | Size (bp) | T(U)% | C% | A% | G% | AT% | GC% | AT skew | GC skew |
| Full genomes | 15415 | 30.6 | 17.4 | 43.1 | 8.9 | 73.7 | 26.3 | 0.1696 | -0.3232 |
| PCGS | 10827 | 42.4 | 14.9 | 29.7 | 12.9 | 72.1 | 27.8 | -0.1761 | -0.0719 |
| tRNAs | 1406 | 35.6 | 10.2 | 39.8 | 14.3 | 75.4 | 24.5 | 0.0557 | 0.1673 |
| rRNAs | 1918 | 46 | 7.4 | 29 | 17.6 | 75 | 25 | -0.2267 | 0.4080 |
| Control region | 1175 | 40.9 | 10.2 | 42 | 6.9 | 82.9 | 17.1 | 0.0133 | -0.1930 |
|  |  |  |  |  |  |  |  |  |  |
| *Andixius truncatus* |  |  |  |  |  |  |  |  |  |
| Regions | Size (bp) | T(U)% | C% | A% | G% | AT% | GC% | AT skew | GC skew |
| Full genomes | 15902 | 31.2 | 16.6 | 43 | 9.2 | 74.2 | 25.8 | 0.1590 | -0.2868 |
| PCGS | 10827 | 42.8 | 14.2 | 30.4 | 12.6 | 73.2 | 26.8 | -0.1694 | -0.0597 |
| tRNAs | 1396 | 35.5 | 10.5 | 39.8 | 14.2 | 75.3 | 24.7 | 0.0571 | 0.1498 |
| rRNAs | 1931 | 46.5 | 8.5 | 27.3 | 17.7 | 73.8 | 26.2 | -0.2602 | 0.3511 |
| Control region | 1650 | 37.7 | 12.1 | 42.6 | 7.6 | 80.3 | 19.7 | 0.0610 | -0.2284 |
|  |  |  |  |  |  |  |  |  |  |
| *Parandes circinatus* |  |  |  |  |  |  |  |  |  |
| Regions | Size (bp) | T(U)% | C% | A% | G% | AT% | GC% | AT skew | GC skew |
| Full genomes | 15445 | 28.5 | 17.6 | 45.2 | 8.7 | 73.7 | 26.3 | 0.2266 | -0.3384 |
| PCGS | 10830 | 41.6 | 15.3 | 30.4 | 12.7 | 72 | 28 | -0.1556 | -0.0929 |
| tRNAs | 1403 | 35.4 | 10.4 | 40.8 | 13.4 | 76.2 | 23.8 | 0.0709 | 0.1261 |
| rRNAs | 1940 | 48.1 | 7.8 | 27.7 | 16.4 | 75.8 | 24.2 | -0.2691 | 0.3554 |
| Control region | 1230 | 35.5 | 11.4 | 46.7 | 6.3 | 82.3 | 17.7 | 0.1361 | -0.2881 |
|  |  |  |  |  |  |  |  |  |  |
| *Parandes fuscus* |  |  |  |  |  |  |  |  |  |
| Regions | Size (bp) | T(U)% | C% | A% | G% | AT% | GC% | AT skew | GC skew |
| Full genomes | 15757 | 30.5 | 15.6 | 45.3 | 8.6 | 75.8 | 24.2 | 0.1953 | -0.2893 |
| PCGS | 10827 | 43.4 | 13.7 | 30.8 | 12.1 | 74.2 | 25.8 | -0.1698 | -0.0620 |
| tRNAs | 1417 | 35.9 | 9.8 | 40.1 | 14.3 | 76 | 24.1 | 0.0553 | 0.1867 |
| rRNAs | 1931 | 47.5 | 7.3 | 29.2 | 16 | 76.7 | 23.3 | -0.2386 | 0.3734 |
| Control region | 1459 | 38.9 | 8.6 | 46.9 | 5.6 | 85.8 | 14.2 | 0.0932 | -0.2113 |

Table S5. Start and stop codons in the mitochondrial genomes of tribe Andini.

| Gene | *A. bifidus* | *A. furcutus* | *A. hemina* | *A. latanalus* | *A. pallidus* | *A. cultratus* | *A. truncatus* | *P. circinatus* | *P. fuscus* |
| --- | --- | --- | --- | --- | --- | --- | --- | --- | --- |
| *nad2* | ATT/TAA | ATT/TAA | ATT/TAA | ATT/TAA | ATT/TAA | ATT/TAA | ATT/TAA | ATT/TAA | ATT/TAA |
| *cox1* | ATT/TAA | ATT/TAA | ATT/TAA | ATT/TAA | ATT/TAA | ATT/TAA | ATT/TAA | ATC/TAA | ATC/TAA |
| *cox2* | ATA/T | ATA/T | ATT/T | ATA/T | ATA/T | ATA/T | ATC/T | ATA/T | ATA/T |
| *atp8* | ATA/TAA | ATA/TAA | ATA/TAA | ATC/TAA | ATC/TAA | ATA/TAA | ATA/TAA | ATA/TAA | ATA/TAA |
| *atp6* | ATA/T | ATA/T | ATT/T | ATA/T | ATT/T | ATT/T | ATT/T | ATA/T | ATT/T |
| *cox3* | ATG/TAA | ATG/TAA | ATG/TAA | ATG/TAA | ATG/TAA | ATG/TAA | ATG/TAA | ATG/TAA | ATG/TAA |
| *nad3* | ATA/TAG | ATA/TAG | ATT/TAA | ATA/TAG | ATT/TAG | ATT/TAG | ATT/TAG | ATA/TAA | ATT/TAA |
| *nad5* | GTG/T | GTG/T | TTG/T | GTG/T | GTG/T | ATC/T | ATT/T | TTG/T | TTG/T |
| *nad4* | ATG/T | ATG/T | ATG/T | ATG/T | ATG/T | ATG/TAA | ATG/TAG | ATG/T | ATG/T |
| *nad4l* | ATG/TAA | ATG/TAA | ATG/TAA | ATG/TAA | ATG/TAA | ATG/TAA | ATG/TAA | ATG/TAA | ATG/TAA |
| *nad6* | ATA/T | ATA/T | ATG/T | ATA/T | ATG/T | ATG/T | ATG/T | ATG/T | ATG/T |
| *cytb* | ATG/TAG | ATG/TAG | ATG/TAG | ATG/TAA | ATG/TAG | ATG/TAG | ATG/TAG | ATG/TAG | ATG/TAG |
| *nad1* | ATG/T | ATG/T | ATG/T | ATG/T | ATG/T | ATG/T | ATG/T | ATG/T | ATG/T |

Table S6. Codon numbers and RSCU in mitochondrial PCGs of Andini species.

| *Andes bifidus* | | | | | | | | | | | |
| --- | --- | --- | --- | --- | --- | --- | --- | --- | --- | --- | --- |
| Codon | Count | RSCU | Codon | Count | RSCU | Codon | Count | RSCU | Codon | Count | RSCU |
| UUU(F) | 396 | 1.75 | UCU(S) | 104 | 2.29 | UAU(Y) | 104 | 1.55 | UGU(C) | 46 | 1.8 |
| UUC(F) | 57 | 0.25 | UCC(S) | 21 | 0.46 | UAC(Y) | 30 | 0.45 | UGC(C) | 5 | 0.2 |
| UUA(L) | 256 | 3.12 | UCA(S) | 126 | 2.77 | UAA(*) | 5 | 1.43 | UGA(W) | 74 | 1.74 |
| UUG(L) | 74 | 0.87 | UCG(S) | 4 | 0.09 | UAG(*) | 2 | 0.57 | UGG(W) | 11 | 0.26 |
| CUU(L) | 70 | 0.82 | CCU(P) | 50 | 1.61 | CAU(H) | 36 | 1.2 | CGU(R) | 16 | 1.25 |
| CUC(L) | 19 | 0.22 | CCC(P) | 25 | 0.81 | CAC(H) | 24 | 0.8 | CGC(R) | 3 | 0.24 |
| CUA(L) | 74 | 0.87 | CCA(P) | 46 | 1.48 | CAA(Q) | 42 | 1.65 | CGA(R) | 26 | 2.04 |
| CUG(L) | 8 | 0.09 | CCG(P) | 3 | 0.1 | CAG(Q) | 9 | 0.35 | CGG(R) | 6 | 0.47 |
| AUU(I) | 322 | 1.69 | ACU(T) | 48 | 1.03 | AAU(N) | 114 | 1.4 | AGU(S) | 26 | 0.57 |
| AUC(I) | 58 | 0.31 | ACC(T) | 39 | 0.83 | AAC(N) | 49 | 0.6 | AGC(S) | 1 | 0.02 |
| AUA(M) | 217 | 1.7 | ACA(T) | 96 | 2.05 | AAA(K) | 130 | 1.9 | AGA(S) | 65 | 1.43 |
| AUG(M) | 39 | 0.3 | ACG(T) | 4 | 0.09 | AAG(K) | 7 | 0.1 | AGG(S) | 17 | 0.37 |
| GUU(V) | 83 | 2.13 | GCU(A) | 34 | 1.32 | GAU(D) | 49 | 1.53 | GGU(G) | 69 | 1.43 |
| GUC(V) | 11 | 0.28 | GCC(A) | 22 | 0.85 | GAC(D) | 15 | 0.47 | GGC(G) | 8 | 0.17 |
| GUA(V) | 50 | 1.28 | GCA(A) | 45 | 1.75 | GAA(E) | 68 | 1.7 | GGA(G) | 82 | 1.7 |
| GUG(V) | 12 | 0.31 | GCG(A) | 2 | 0.08 | GAG(E) | 12 | 0.3 | GGG(G) | 34 | 0.7 |
| Average# codons=3609 | | |  |  |  |  |  |  |  |  |  |
|  |  |  |  |  |  |  |  |  |  |  |  |
| *Andes furcutus* | | | | | | | | | | | |
| Codon | Count | RSCU | Codon | Count | RSCU | Codon | Count | RSCU | Codon | Count | RSCU |
| UUU(F) | 397 | 1.72 | UCU(S) | 97 | 2.05 | UAU(Y) | 110 | 1.62 | UGU(C) | 44 | 1.76 |
| UUC(F) | 64 | 0.28 | UCC(S) | 39 | 0.83 | UAC(Y) | 26 | 0.38 | UGC(C) | 6 | 0.24 |
| UUA(L) | 239 | 2.86 | UCA(S) | 118 | 2.5 | UAA(*) | 5 | 1.43 | UGA(W) | 74 | 1.78 |
| UUG(L) | 73 | 0.87 | UCG(S) | 5 | 0.11 | UAG(*) | 2 | 0.57 | UGG(W) | 9 | 0.22 |
| CUU(L) | 74 | 0.89 | CCU(P) | 53 | 1.74 | CAU(H) | 32 | 1.05 | CGU(R) | 17 | 1.36 |
| CUC(L) | 17 | 0.2 | CCC(P) | 29 | 0.95 | CAC(H) | 29 | 0.95 | CGC(R) | 3 | 0.24 |
| CUA(L) | 88 | 1.05 | CCA(P) | 35 | 1.15 | CAA(Q) | 44 | 1.66 | CGA(R) | 26 | 2.08 |
| CUG(L) | 10 | 0.12 | CCG(P) | 5 | 0.16 | CAG(Q) | 9 | 0.34 | CGG(R) | 4 | 0.32 |
| AUU(I) | 291 | 1.6 | ACU(T) | 62 | 1.27 | AAU(N) | 111 | 1.39 | AGU(S) | 31 | 0.66 |
| AUC(I) | 72 | 0.4 | ACC(T) | 35 | 0.72 | AAC(N) | 49 | 0.61 | AGC(S) | 4 | 0.08 |
| AUA(M) | 222 | 1.72 | ACA(T) | 93 | 1.91 | AAA(K) | 114 | 1.74 | AGA(S) | 62 | 1.31 |
| AUG(M) | 36 | 0.28 | ACG(T) | 5 | 0.1 | AAG(K) | 17 | 0.26 | AGG(S) | 22 | 0.47 |
| GUU(V) | 85 | 2.18 | GCU(A) | 38 | 1.42 | GAU(D) | 48 | 1.43 | GGU(G) | 81 | 1.64 |
| GUC(V) | 7 | 0.18 | GCC(A) | 28 | 1.05 | GAC(D) | 19 | 0.57 | GGC(G) | 15 | 0.3 |
| GUA(V) | 53 | 1.36 | GCA(A) | 38 | 1.42 | GAA(E) | 60 | 1.6 | GGA(G) | 73 | 1.48 |
| GUG(V) | 11 | 0.28 | GCG(A) | 3 | 0.11 | GAG(E) | 15 | 0.4 | GGG(G) | 28 | 0.57 |
| Average# codons=3611 | | |  |  |  |  |  |  |  |  |  |
|  |  |  |  |  |  |  |  |  |  |  |  |
| *Andes hemina* | | | | | | | | | | | |
| Codon | Count | RSCU | Codon | Count | RSCU | Codon | Count | RSCU | Codon | Count | RSCU |
| UUU(F) | 408 | 1.74 | UCU(S) | 103 | 2.09 | UAU(Y) | 94 | 1.41 | UGU(C) | 43 | 1.76 |
| UUC(F) | 60 | 0.26 | UCC(S) | 25 | 0.51 | UAC(Y) | 39 | 0.59 | UGC(C) | 6 | 0.24 |
| UUA(L) | 249 | 3.08 | UCA(S) | 133 | 2.69 | UAA(*) | 6 | 1.71 | UGA(W) | 77 | 1.75 |
| UUG(L) | 75 | 0.93 | UCG(S) | 11 | 0.22 | UAG(*) | 1 | 0.29 | UGG(W) | 11 | 0.25 |
| CUU(L) | 75 | 0.93 | CCU(P) | 45 | 1.38 | CAU(H) | 32 | 1.03 | CGU(R) | 15 | 1.2 |
| CUC(L) | 7 | 0.09 | CCC(P) | 15 | 0.46 | CAC(H) | 30 | 0.97 | CGC(R) | 0 | 0 |
| CUA(L) | 67 | 0.83 | CCA(P) | 65 | 2 | CAA(Q) | 43 | 1.59 | CGA(R) | 27 | 2.16 |
| CUG(L) | 12 | 0.15 | CCG(P) | 5 | 0.15 | CAG(Q) | 11 | 0.41 | CGG(R) | 8 | 0.64 |
| AUU(I) | 306 | 1.66 | ACU(T) | 56 | 1.18 | AAU(N) | 92 | 1.25 | AGU(S) | 31 | 0.63 |
| AUC(I) | 63 | 0.34 | ACC(T) | 34 | 0.72 | AAC(N) | 55 | 0.75 | AGC(S) | 5 | 0.1 |
| AUA(M) | 210 | 1.67 | ACA(T) | 99 | 2.08 | AAA(K) | 115 | 1.73 | AGA(S) | 75 | 1.52 |
| AUG(M) | 42 | 0.33 | ACG(T) | 1 | 0.02 | AAG(K) | 18 | 0.27 | AGG(S) | 12 | 0.24 |
| GUU(V) | 89 | 2.3 | GCU(A) | 42 | 1.57 | GAU(D) | 38 | 1.27 | GGU(G) | 80 | 1.64 |
| GUC(V) | 7 | 0.18 | GCC(A) | 17 | 0.64 | GAC(D) | 22 | 0.73 | GGC(G) | 12 | 0.25 |
| GUA(V) | 50 | 1.29 | GCA(A) | 45 | 1.68 | GAA(E) | 61 | 1.52 | GGA(G) | 71 | 1.46 |
| GUG(V) | 9 | 0.23 | GCG(A) | 3 | 0.11 | GAG(E) | 19 | 0.47 | GGG(G) | 32 | 0.66 |
| Average# codons=3609 | | |  |  |  |  |  |  |  |  |  |
|  |  |  |  |  |  |  |  |  |  |  |  |
| *Andes latanalus* | | | | | | | | | | | |
| Codon | Count | RSCU | Codon | Count | RSCU | Codon | Count | RSCU | Codon | Count | RSCU |
| UUU(F) | 401 | 1.77 | UCU(S) | 105 | 2.21 | UAU(Y) | 110 | 1.62 | UGU(C) | 47 | 1.81 |
| UUC(F) | 51 | 0.23 | UCC(S) | 33 | 0.69 | UAC(Y) | 26 | 0.38 | UGC(C) | 5 | 0.19 |
| UUA(L) | 244 | 2.9 | UCA(S) | 118 | 2.48 | UAA(*) | 6 | 1.71 | UGA(W) | 74 | 1.74 |
| UUG(L) | 82 | 0.98 | UCG(S) | 3 | 0.06 | UAG(*) | 1 | 0.29 | UGG(W) | 11 | 0.26 |
| CUU(L) | 75 | 0.89 | CCU(P) | 39 | 1.25 | CAU(H) | 38 | 1.27 | CGU(R) | 17 | 1.36 |
| CUC(L) | 17 | 0.2 | CCC(P) | 35 | 1.12 | CAC(H) | 22 | 0.73 | CGC(R) | 0 | 0 |
| CUA(L) | 79 | 0.94 | CCA(P) | 48 | 1.54 | CAA(Q) | 41 | 1.52 | CGA(R) | 29 | 2.32 |
| CUG(L) | 7 | 0.08 | CCG(P) | 3 | 0.1 | CAG(Q) | 13 | 0.48 | CGG(R) | 4 | 0.32 |
| AUU(I) | 281 | 1.54 | ACU(T) | 54 | 1.13 | AAU(N) | 109 | 1.35 | AGU(S) | 29 | 0.61 |
| AUC(I) | 85 | 0.46 | ACC(T) | 43 | 0.9 | AAC(N) | 52 | 0.65 | AGC(S) | 0 | 0 |
| AUA(M) | 219 | 1.67 | ACA(T) | 89 | 1.86 | AAA(K) | 120 | 1.85 | AGA(S) | 69 | 1.45 |
| AUG(M) | 44 | 0.33 | ACG(T) | 5 | 0.1 | AAG(K) | 10 | 0.15 | AGG(S) | 23 | 0.48 |
| GUU(V) | 71 | 1.86 | GCU(A) | 40 | 1.57 | GAU(D) | 50 | 1.54 | GGU(G) | 81 | 1.63 |
| GUC(V) | 9 | 0.24 | GCC(A) | 18 | 0.71 | GAC(D) | 15 | 0.46 | GGC(G) | 7 | 0.14 |
| GUA(V) | 63 | 1.65 | GCA(A) | 39 | 1.53 | GAA(E) | 58 | 1.57 | GGA(G) | 84 | 1.69 |
| GUG(V) | 10 | 0.26 | GCG(A) | 5 | 0.2 | GAG(E) | 16 | 0.43 | GGG(G) | 27 | 0.54 |
| Average# codons=3609 | | |  |  |  |  |  |  |  |  |  |
|  |  |  |  |  |  |  |  |  |  |  |  |
| *Andes pallidus* | | | | | | | | | | | |
| Codon | Count | RSCU | Codon | Count | RSCU | Codon | Count | RSCU | Codon | Count | RSCU |
| UUU(F) | 400 | 1.75 | UCU(S) | 103 | 2.09 | UAU(Y) | 122 | 1.81 | UGU(C) | 45 | 1.84 |
| UUC(F) | 58 | 0.25 | UCC(S) | 31 | 0.63 | UAC(Y) | 13 | 0.19 | UGC(C) | 4 | 0.16 |
| UUA(L) | 299 | 3.53 | UCA(S) | 134 | 2.72 | UAA(*) | 5 | 1.43 | UGA(W) | 76 | 1.77 |
| UUG(L) | 51 | 0.6 | UCG(S) | 2 | 0.04 | UAG(*) | 2 | 0.57 | UGG(W) | 10 | 0.23 |
| CUU(L) | 73 | 0.86 | CCU(P) | 50 | 1.6 | CAU(H) | 31 | 1.02 | CGU(R) | 13 | 1.04 |
| CUC(L) | 21 | 0.25 | CCC(P) | 23 | 0.74 | CAC(H) | 30 | 0.98 | CGC(R) | 3 | 0.24 |
| CUA(L) | 63 | 0.74 | CCA(P) | 50 | 1.6 | CAA(Q) | 45 | 1.64 | CGA(R) | 29 | 2.32 |
| CUG(L) | 1 | 0.01 | CCG(P) | 2 | 0.06 | CAG(Q) | 10 | 0.36 | CGG(R) | 5 | 0.4 |
| AUU(I) | 314 | 1.66 | ACU(T) | 45 | 1.04 | AAU(N) | 121 | 1.49 | AGU(S) | 42 | 0.85 |
| AUC(I) | 65 | 0.34 | ACC(T) | 44 | 1.02 | AAC(N) | 41 | 0.51 | AGC(S) | 2 | 0.04 |
| AUA(M) | 239 | 1.83 | ACA(T) | 83 | 1.92 | AAA(K) | 119 | 1.84 | AGA(S) | 62 | 1.26 |
| AUG(M) | 22 | 0.17 | ACG(T) | 1 | 0.02 | AAG(K) | 10 | 0.16 | AGG(S) | 18 | 0.37 |
| GUU(V) | 82 | 2.2 | GCU(A) | 49 | 2.06 | GAU(D) | 48 | 1.5 | GGU(G) | 67 | 1.38 |
| GUC(V) | 7 | 0.19 | GCC(A) | 11 | 0.46 | GAC(D) | 16 | 0.5 | GGC(G) | 4 | 0.08 |
| GUA(V) | 53 | 1.42 | GCA(A) | 34 | 1.43 | GAA(E) | 65 | 1.73 | GGA(G) | 89 | 1.84 |
| GUG(V) | 7 | 0.19 | GCG(A) | 1 | 0.04 | GAG(E) | 10 | 0.27 | GGG(G) | 34 | 0.7 |
| Average# codons=3609 | | |  |  |  |  |  |  |  |  |  |
|  |  |  |  |  |  |  |  |  |  |  |  |
| *Andixius cultratus* | | | | | | | | | | | |
| Codon | Count | RSCU | Codon | Count | RSCU | Codon | Count | RSCU | Codon | Count | RSCU |
| UUU(F) | 384 | 1.7 | UCU(S) | 98 | 1.96 | UAU(Y) | 114 | 1.62 | UGU(C) | 38 | 1.69 |
| UUC(F) | 67 | 0.3 | UCC(S) | 38 | 0.76 | UAC(Y) | 27 | 0.38 | UGC(C) | 7 | 0.31 |
| UUA(L) | 244 | 2.84 | UCA(S) | 123 | 2.45 | UAA(*) | 6 | 1.5 | UGA(W) | 67 | 1.63 |
| UUG(L) | 86 | 1 | UCG(S) | 8 | 0.16 | UAG(*) | 2 | 0.5 | UGG(W) | 15 | 0.37 |
| CUU(L) | 62 | 0.72 | CCU(P) | 33 | 1.04 | CAU(H) | 36 | 1.2 | CGU(R) | 17 | 1.39 |
| CUC(L) | 25 | 0.29 | CCC(P) | 46 | 1.45 | CAC(H) | 24 | 0.8 | CGC(R) | 3 | 0.24 |
| CUA(L) | 83 | 0.97 | CCA(P) | 47 | 1.48 | CAA(Q) | 42 | 1.53 | CGA(R) | 25 | 2.04 |
| CUG(L) | 15 | 0.17 | CCG(P) | 1 | 0.03 | CAG(Q) | 13 | 0.47 | CGG(R) | 4 | 0.33 |
| AUU(I) | 291 | 1.59 | ACU(T) | 53 | 1.17 | AAU(N) | 117 | 1.49 | AGU(S) | 39 | 0.78 |
| AUC(I) | 74 | 0.41 | ACC(T) | 41 | 0.91 | AAC(N) | 40 | 0.51 | AGC(S) | 4 | 0.08 |
| AUA(M) | 213 | 1.67 | ACA(T) | 82 | 1.81 | AAA(K) | 105 | 1.72 | AGA(S) | 71 | 1.42 |
| AUG(M) | 42 | 0.33 | ACG(T) | 5 | 0.11 | AAG(K) | 17 | 0.28 | AGG(S) | 20 | 0.4 |
| GUU(V) | 85 | 2.09 | GCU(A) | 37 | 1.54 | GAU(D) | 46 | 1.35 | GGU(G) | 71 | 1.45 |
| GUC(V) | 13 | 0.32 | GCC(A) | 25 | 1.04 | GAC(D) | 22 | 0.65 | GGC(G) | 13 | 0.27 |
| GUA(V) | 52 | 1.28 | GCA(A) | 32 | 1.33 | GAA(E) | 56 | 1.56 | GGA(G) | 76 | 1.55 |
| GUG(V) | 13 | 0.32 | GCG(A) | 2 | 0.08 | GAG(E) | 16 | 0.44 | GGG(G) | 36 | 0.73 |
| Average# codons=3609 | | |  |  |  |  |  |  |  |  |  |
|  |  |  |  |  |  |  |  |  |  |  |  |
| *Andixius truncatus* | | | | | | | | | | | |
| Codon | Count | RSCU | Codon | Count | RSCU | Codon | Count | RSCU | Codon | Count | RSCU |
| UUU(F) | 383 | 1.71 | UCU(S) | 99 | 1.99 | UAU(Y) | 114 | 1.62 | UGU(C) | 44 | 1.96 |
| UUC(F) | 65 | 0.29 | UCC(S) | 39 | 0.78 | UAC(Y) | 27 | 0.38 | UGC(C) | 1 | 0.04 |
| UUA(L) | 258 | 3.01 | UCA(S) | 124 | 2.49 | UAA(*) | 5 | 1.25 | UGA(W) | 69 | 1.66 |
| UUG(L) | 80 | 0.93 | UCG(S) | 7 | 0.14 | UAG(*) | 3 | 0.75 | UGG(W) | 14 | 0.34 |
| CUU(L) | 68 | 0.79 | CCU(P) | 41 | 1.32 | CAU(H) | 35 | 1.17 | CGU(R) | 15 | 1.28 |
| CUC(L) | 12 | 0.14 | CCC(P) | 34 | 1.1 | CAC(H) | 25 | 0.83 | CGC(R) | 4 | 0.34 |
| CUA(L) | 83 | 0.97 | CCA(P) | 47 | 1.52 | CAA(Q) | 39 | 1.39 | CGA(R) | 26 | 2.21 |
| CUG(L) | 13 | 0.15 | CCG(P) | 2 | 0.06 | CAG(Q) | 17 | 0.61 | CGG(R) | 2 | 0.17 |
| AUU(I) | 315 | 1.68 | ACU(T) | 50 | 1.12 | AAU(N) | 115 | 1.42 | AGU(S) | 30 | 0.6 |
| AUC(I) | 61 | 0.32 | ACC(T) | 38 | 0.85 | AAC(N) | 47 | 0.58 | AGC(S) | 4 | 0.08 |
| AUA(M) | 220 | 1.71 | ACA(T) | 88 | 1.97 | AAA(K) | 105 | 1.71 | AGA(S) | 72 | 1.45 |
| AUG(M) | 38 | 0.29 | ACG(T) | 3 | 0.07 | AAG(K) | 18 | 0.29 | AGG(S) | 23 | 0.46 |
| GUU(V) | 81 | 2.08 | GCU(A) | 41 | 1.78 | GAU(D) | 52 | 1.55 | GGU(G) | 64 | 1.31 |
| GUC(V) | 9 | 0.23 | GCC(A) | 17 | 0.74 | GAC(D) | 15 | 0.45 | GGC(G) | 12 | 0.24 |
| GUA(V) | 57 | 1.46 | GCA(A) | 32 | 1.39 | GAA(E) | 63 | 1.66 | GGA(G) | 86 | 1.76 |
| GUG(V) | 9 | 0.23 | GCG(A) | 2 | 0.09 | GAG(E) | 13 | 0.34 | GGG(G) | 34 | 0.69 |
| Average# codons=3609 | | |  |  |  |  |  |  |  |  |  |
|  |  |  |  |  |  |  |  |  |  |  |  |
| *Parandes circinatus* | | | | | | | | | | | |
| Codon | Count | RSCU | Codon | Count | RSCU | Codon | Count | RSCU | Codon | Count | RSCU |
| UUU(F) | 381 | 1.63 | UCU(S) | 92 | 1.92 | UAU(Y) | 105 | 1.59 | UGU(C) | 44 | 1.83 |
| UUC(F) | 86 | 0.37 | UCC(S) | 35 | 0.73 | UAC(Y) | 27 | 0.41 | UGC(C) | 4 | 0.17 |
| UUA(L) | 216 | 2.73 | UCA(S) | 126 | 2.62 | UAA(*) | 6 | 1.71 | UGA(W) | 73 | 1.7 |
| UUG(L) | 80 | 1.01 | UCG(S) | 8 | 0.17 | UAG(*) | 1 | 0.29 | UGG(W) | 13 | 0.3 |
| CUU(L) | 71 | 0.9 | CCU(P) | 44 | 1.38 | CAU(H) | 31 | 1 | CGU(R) | 20 | 1.6 |
| CUC(L) | 16 | 0.2 | CCC(P) | 30 | 0.94 | CAC(H) | 31 | 1 | CGC(R) | 0 | 0 |
| CUA(L) | 86 | 1.09 | CCA(P) | 54 | 1.69 | CAA(Q) | 48 | 1.66 | CGA(R) | 26 | 2.08 |
| CUG(L) | 6 | 0.08 | CCG(P) | 0 | 0 | CAG(Q) | 10 | 0.34 | CGG(R) | 4 | 0.32 |
| AUU(I) | 268 | 1.43 | ACU(T) | 56 | 1.13 | AAU(N) | 95 | 1.28 | AGU(S) | 31 | 0.65 |
| AUC(I) | 108 | 0.57 | ACC(T) | 34 | 0.68 | AAC(N) | 54 | 0.72 | AGC(S) | 5 | 0.1 |
| AUA(M) | 225 | 1.67 | ACA(T) | 106 | 2.13 | AAA(K) | 112 | 1.76 | AGA(S) | 68 | 1.42 |
| AUG(M) | 44 | 0.33 | ACG(T) | 3 | 0.06 | AAG(K) | 15 | 0.24 | AGG(S) | 19 | 0.4 |
| GUU(V) | 79 | 2.11 | GCU(A) | 34 | 1.35 | GAU(D) | 41 | 1.32 | GGU(G) | 78 | 1.51 |
| GUC(V) | 10 | 0.27 | GCC(A) | 23 | 0.91 | GAC(D) | 21 | 0.68 | GGC(G) | 8 | 0.16 |
| GUA(V) | 49 | 1.31 | GCA(A) | 40 | 1.58 | GAA(E) | 63 | 1.7 | GGA(G) | 85 | 1.65 |
| GUG(V) | 12 | 0.32 | GCG(A) | 4 | 0.16 | GAG(E) | 11 | 0.3 | GGG(G) | 35 | 0.68 |
| Average# codons=3610 | | |  |  |  |  |  |  |  |  |  |
|  |  |  |  |  |  |  |  |  |  |  |  |
| *Parandes fuscus* | | | | | | | | | | | |
| Codon | Count | RSCU | Codon | Count | RSCU | Codon | Count | RSCU | Codon | Count | RSCU |
| UUU(F) | 418 | 1.77 | UCU(S) | 111 | 2.29 | UAU(Y) | 112 | 1.66 | UGU(C) | 42 | 1.91 |
| UUC(F) | 55 | 0.23 | UCC(S) | 21 | 0.43 | UAC(Y) | 23 | 0.34 | UGC(C) | 2 | 0.09 |
| UUA(L) | 266 | 3.35 | UCA(S) | 135 | 2.78 | UAA(*) | 6 | 1.71 | UGA(W) | 73 | 1.68 |
| UUG(L) | 60 | 0.76 | UCG(S) | 4 | 0.08 | UAG(*) | 1 | 0.29 | UGG(W) | 14 | 0.32 |
| CUU(L) | 65 | 0.82 | CCU(P) | 55 | 1.71 | CAU(H) | 46 | 1.46 | CGU(R) | 24 | 1.92 |
| CUC(L) | 13 | 0.16 | CCC(P) | 20 | 0.62 | CAC(H) | 17 | 0.54 | CGC(R) | 0 | 0 |
| CUA(L) | 64 | 0.81 | CCA(P) | 48 | 1.49 | CAA(Q) | 45 | 1.67 | CGA(R) | 25 | 2 |
| CUG(L) | 8 | 0.1 | CCG(P) | 6 | 0.19 | CAG(Q) | 9 | 0.33 | CGG(R) | 1 | 0.08 |
| AUU(I) | 308 | 1.65 | ACU(T) | 55 | 1.18 | AAU(N) | 93 | 1.27 | AGU(S) | 32 | 0.66 |
| AUC(I) | 65 | 0.35 | ACC(T) | 30 | 0.64 | AAC(N) | 53 | 0.73 | AGC(S) | 4 | 0.08 |
| AUA(M) | 232 | 1.74 | ACA(T) | 102 | 2.18 | AAA(K) | 122 | 1.83 | AGA(S) | 66 | 1.36 |
| AUG(M) | 34 | 0.26 | ACG(T) | 0 | 0 | AAG(K) | 11 | 0.17 | AGG(S) | 15 | 0.31 |
| GUU(V) | 89 | 2.33 | GCU(A) | 36 | 1.44 | GAU(D) | 39 | 1.26 | GGU(G) | 90 | 1.77 |
| GUC(V) | 5 | 0.13 | GCC(A) | 13 | 0.52 | GAC(D) | 23 | 0.74 | GGC(G) | 8 | 0.16 |
| GUA(V) | 51 | 1.33 | GCA(A) | 50 | 2 | GAA(E) | 68 | 1.7 | GGA(G) | 78 | 1.54 |
| GUG(V) | 8 | 0.21 | GCG(A) | 1 | 0.04 | GAG(E) | 12 | 0.3 | GGG(G) | 27 | 0.53 |
| Average# codons=3609 | | |  |  |  |  |  |  |  |  |  |

Table S7. AT and GC skews of 13 PCGs across 16 Cixiidae mitogenomes

| AT skew | | | | | | | | | | | | | | | | |
| --- | --- | --- | --- | --- | --- | --- | --- | --- | --- | --- | --- | --- | --- | --- | --- | --- |
| Gene | *AB* | *AF* | *AH* | *AL* | *AP* | *AC* | *AT* | *PC* | *PF* | *BS* | CS | CIS | *IP* | *OS* | *OCS* | *OF* |
| *atp6* | 0.048 | 0.098 | 0.059 | 0.081 | 0.037 | 0.034 | 0.048 | 0.089 | 0.036 | 0.154 | 0.039 | 0.016 | 0.120 | 0.068 | 0.070 | 0.053 |
| *atp8* | 0.079 | 0.013 | 0.025 | 0.091 | 0.075 | 0.067 | 0.038 | 0.026 | 0.012 | 0.263 | 0.068 | 0.047 | 0.258 | 0.371 | 0.371 | 0.136 |
| *cox1* | 0.022 | 0.011 | 0.027 | 0.014 | 0.011 | 0.003 | -0.005 | 0.020 | 0.012 | 0.110 | -0.036 | -0.027 | 0.059 | 0.015 | 0.009 | 0.019 |
| *cox2* | 0.163 | 0.166 | 0.171 | 0.167 | 0.105 | 0.096 | 0.063 | 0.200 | 0.157 | 0.231 | 0.085 | 0.069 | 0.204 | 0.153 | 0.165 | 0.125 |
| *cox3* | 0.094 | 0.074 | 0.092 | 0.096 | 0.066 | 0.114 | 0.051 | 0.160 | 0.093 | 0.166 | 0.079 | 0.034 | 0.121 | 0.080 | 0.085 | 0.087 |
| *cytb* | -0.070 | -0.097 | -0.063 | -0.064 | -0.078 | -0.096 | -0.090 | -0.005 | -0.055 | 0.084 | -0.069 | -0.067 | 0.006 | 0.022 | -0.026 | -0.029 |
| *nad1* | -0.380 | -0.420 | -0.429 | -0.404 | -0.385 | -0.374 | -0.371 | -0.449 | -0.425 | -0.502 | -0.361 | -0.392 | -0.427 | -0.426 | -0.407 | -0.367 |
| *nad2* | 0.090 | 0.090 | 0.072 | 0.127 | 0.080 | 0.030 | 0.012 | 0.141 | 0.069 | 0.242 | 0.083 | 0.072 | 0.142 | 0.112 | 0.114 | 0.107 |
| *nad3* | -0.020 | 0.023 | 0.045 | 0.008 | 0.008 | -0.004 | -0.023 | 0.067 | 0.031 | 0.116 | 0.004 | 0.000 | 0.092 | 0.000 | 0.018 | -0.029 |
| *nad4* | -0.470 | -0.487 | -0.488 | -0.486 | -0.461 | -0.469 | -0.429 | -0.463 | -0.476 | -0.530 | -0.377 | -0.380 | -0.454 | -0.467 | -0.434 | -0.391 |
| *nad4l* | -0.495 | -0.526 | -0.456 | -0.577 | -0.521 | -0.463 | -0.330 | -0.555 | -0.523 | -0.551 | -0.368 | -0.479 | -0.482 | -0.472 | -0.496 | -0.434 |
| *nad5* | -0.489 | -0.512 | -0.501 | -0.524 | -0.466 | -0.498 | -0.457 | -0.529 | -0.502 | -0.545 | -0.462 | -0.395 | -0.510 | -0.446 | -0.458 | -0.397 |
| *nad6* | -0.046 | 0.011 | -0.062 | 0.010 | -0.043 | -0.034 | -0.056 | -0.028 | -0.025 | 0.205 | 0.025 | -0.052 | 0.094 | 0.087 | 0.079 | 0.049 |
|  |  |  |  |  |  |  |  |  |  |  |  |  |  |  |  |  |
| GC skew | | | | | | | | | | | | | | | | |
| Gene | *AB* | *AF* | *AH* | *AL* | *AP* | *AC* | *AT* | *PC* | *PF* | *BS* | CS | CIS | *IP* | *OS* | *OCS* | *OF* |
| *atp6* | -0.407 | -0.500 | -0.436 | -0.421 | -0.448 | -0.503 | -0.442 | -0.530 | -0.453 | -0.550 | -0.319 | -0.409 | -0.424 | -0.438 | -0.447 | -0.397 |
| *atp8* | -0.769 | -0.565 | -0.636 | -0.680 | -0.818 | -0.778 | -0.739 | -0.750 | -0.714 | -0.692 | -0.700 | -0.455 | -0.625 | -0.625 | -0.625 | -0.765 |
| *cox1* | -0.170 | -0.201 | -0.151 | -0.180 | -0.190 | -0.244 | -0.203 | -0.222 | -0.161 | -0.263 | -0.075 | -0.124 | -0.146 | -0.096 | -0.091 | -0.079 |
| *cox2* | -0.407 | -0.401 | -0.310 | -0.426 | -0.388 | -0.235 | -0.275 | -0.333 | -0.337 | -0.451 | -0.176 | -0.251 | -0.276 | -0.161 | -0.185 | -0.278 |
| *cox3* | -0.200 | -0.195 | -0.169 | -0.193 | -0.255 | -0.260 | -0.165 | -0.292 | -0.202 | -0.358 | -0.103 | -0.144 | -0.175 | -0.121 | -0.127 | -0.110 |
| *cytb* | -0.248 | -0.274 | -0.218 | -0.300 | -0.262 | -0.241 | -0.245 | -0.296 | -0.238 | -0.382 | -0.148 | -0.259 | -0.244 | -0.228 | -0.240 | -0.256 |
| *nad1* | 0.324 | 0.339 | 0.352 | 0.360 | 0.327 | 0.310 | 0.328 | 0.350 | 0.322 | 0.388 | 0.211 | 0.228 | 0.399 | 0.222 | 0.237 | 0.252 |
| *nad2* | -0.493 | -0.527 | -0.470 | -0.619 | -0.589 | -0.487 | -0.493 | -0.509 | -0.498 | -0.612 | -0.476 | -0.457 | -0.513 | -0.393 | -0.436 | -0.438 |
| *nad3* | -0.306 | -0.411 | -0.264 | -0.368 | -0.320 | -0.320 | -0.187 | -0.375 | -0.281 | -0.400 | -0.237 | -0.247 | -0.217 | -0.231 | -0.289 | -0.315 |
| *nad4* | 0.418 | 0.380 | 0.321 | 0.397 | 0.397 | 0.386 | 0.326 | 0.383 | 0.378 | 0.367 | 0.154 | 0.218 | 0.339 | 0.192 | 0.184 | 0.302 |
| *nad4l* | 0.690 | 0.631 | 0.424 | 0.524 | 0.661 | 0.662 | 0.574 | 0.477 | 0.517 | 0.558 | 0.556 | 0.344 | 0.660 | 0.455 | 0.440 | 0.560 |
| *nad5* | 0.353 | 0.368 | 0.270 | 0.438 | 0.392 | 0.362 | 0.322 | 0.378 | 0.296 | 0.350 | 0.220 | 0.219 | 0.295 | 0.210 | 0.240 | 0.310 |
| *nad6* | -0.423 | -0.466 | -0.226 | -0.358 | -0.379 | -0.415 | -0.340 | -0.354 | -0.271 | -0.463 | -0.308 | -0.437 | -0.400 | -0.402 | -0.461 | -0.358 |

Notes: AB (*Andes bifidus*), AF (*Andes furcutus*), AH (*Andes hemina*), AL (*Andes latanalus*), AP (*Andes pallidus*), AC (*Andixius cultratus*), AT (*Andixius truncates*), PC (*Parandes circinatus*), PF (*Parandes fuscus*), BS (*Borysthenes* sp.), CS (Cixiidae sp.), CIS (Cixiini sp.), IP (*Iolania perkinsi*), OS (*Oecleopsis sinicus*), OCS (*Oecleopsis* sp. ), OF (*Oliarus filicicola*).

TableS8. The nucleotide diversity values of 13 PCGs in Andini

| *atp6* | *atp8* | *cox1* | *cox2* | *cox3* | *cytb* | *nad1* | *nad2* | *nad3* | *nad4* | *nad4l* | *nad5* | *nad6* |
| --- | --- | --- | --- | --- | --- | --- | --- | --- | --- | --- | --- | --- |
| 0.2201 | 0.2553 | 0.1646 | 0.2183 | 0.2076 | 0.1918 | 0.1809 | 0.2659 | 0.2370 | 0.1788 | 0.1894 | 0.1803 | 0.2655 |

Table S9. The Ka, Ks, and Ka/Ks values for the 13 PCGs of the nine Andini species.

|  | *atp6* | *atp8* | *cox1* | *cox2* | *cox3* | *cytb* | *nad1* | *nad2* | *nad3* | *nad4* | *nad4l* | *nad5* | *nad6* |
| --- | --- | --- | --- | --- | --- | --- | --- | --- | --- | --- | --- | --- | --- |
| Ka | 0.1331 | 0.1927 | 0.0505 | 0.1250 | 0.1304 | 0.0856 | 0.1271 | 0.2168 | 0.1496 | 0.1432 | 0.1562 | 0.1478 | 0.2274 |
| Ks | 0.9373 | 1.2367 | 0.8730 | 1.1440 | 0.8470 | 1.0159 | 0.5897 | 0.9394 | 1.1449 | 0.4781 | 0.5344 | 0.4799 | 0.8983 |
| Ka/Ks | 0.1420 | 0.1558 | 0.0578 | 0.1092 | 0.1539 | 0.0843 | 0.2156 | 0.2308 | 0.1306 | 0.2995 | 0.2922 | 0.3080 | 0.2531 |
